# Early physiological phenotypes and survival outcomes in advanced non-small cell lung cancer: a longitudinal study of dynamic muscle loss and functional decline

**DOI:** 10.3389/fonc.2026.1882281

**Published:** 2026-08-14

**Authors:** Liang Yan, Yongcun Wu, Xi Jiang, Xuepin Yao

**Affiliations:** Department of Radiotherapy and Chemotherapy, The Second Affiliated Hospital of Xingtai Medical College, Xingtai, Hebei, China

**Keywords:** immuno-chemotherapy, non-small cell lung cancer, overall survival, physiological phenotype, prognosis, sarcopenia

## Abstract

**Objective:**

To characterize early changes in skeletal muscle and physical function among patients with advanced non-small cell lung cancer (NSCLC) receiving penpulimab monotherapy or combination therapy, and to identify distinct treatment-associated physiological phenotypes with potential clinical relevance.

**Methods:**

We retrospectively analyzed 381 patients with advanced NSCLC (monotherapy, *n* = 153; combination therapy, *n* = 228) treated between September 2021 and March 2023. Inverse probability of treatment weighting (IPTW) was applied to balance baseline characteristics between treatment groups. Skeletal muscle index (SMI), muscle attenuation, and functional measures were assessed at baseline and 12 weeks. Unsupervised clustering was performed to identify early physiological phenotypes based on longitudinal changes in muscle and functional parameters. Exploratory survival analyses were conducted using 12-week landmark models to evaluate the association between physiological phenotypes and overall survival (OS).

**Results:**

After IPTW adjustment, combination therapy was associated with greater early physiological deterioration, including larger declines in muscle mass and physical performance, increased incidence of new-onset sarcopenia, and poorer treatment tolerability compared with monotherapy. Unsupervised clustering identified three distinct physiological phenotypes: Stable (P1), Wasting (P2), and Collapse (P3). Combination therapy was associated with a higher likelihood of transition to vulnerable phenotypes, particularly P2 and P3. These phenotypes demonstrated distinct clinical characteristics and survival patterns, with the Collapse phenotype exhibiting the poorest OS (median OS, 18.0 months), whereas OS was not reached in the Stable phenotype. Incorporation of physiological phenotypes improved the discrimination of landmark survival models beyond baseline clinical characteristics and radiographic response assessments. Exploratory pathway analyses suggested potential associations between early physiological deterioration patterns and survival differences; however, these findings require prospective validation.

**Conclusions:**

Early treatment-associated physiological deterioration in advanced NSCLC is heterogeneous and can be characterized by reproducible dynamic phenotypes. Combination therapy is associated with greater early declines in muscle and physical function, while longitudinal physiological phenotyping provides complementary information for identifying patients at increased risk of adverse outcomes beyond conventional tumor response assessment. Prospective studies are warranted to validate these findings and determine whether interventions targeting physiological decline can improve clinical outcomes.

## 1 Introduction

The introduction of immune checkpoint inhibitors (ICIs) has substantially transformed the therapeutic landscape of advanced non-small cell lung cancer (NSCLC), resulting in meaningful improvements in survival outcomes for selected patients (1–3). Penpulimab, a novel PD-1 inhibitor developed in China, has demonstrated favorable antitumor activity and clinical benefit both as monotherapy and in combination with chemotherapy (4). However, despite their superior antitumor efficacy, intensified treatment regimens are frequently accompanied by increased systemic toxicity, impaired physical function, and early physiological deterioration (5, 6). Consequently, balancing treatment efficacy with treatment-related physiological burden has become an increasingly critical challenge in the management of advanced NSCLC.

Sarcopenia and impaired physical function are well-established predictors of adverse outcomes in patients with advanced malignancies (7). In the setting of immuno-chemotherapy, treatment-induced metabolic disturbances and systemic inflammatory responses may accelerate declines in skeletal muscle and physical performance (8, 9). Nevertheless, previous studies have largely focused on baseline measures of body composition or functional status, whereas the dynamic evolution of physiological changes during treatment has been considerably less investigated. In particular, it remains unclear whether early treatment-related physiological changes follow distinct trajectories and whether such heterogeneity provides prognostic information beyond conventional tumor response assessments.

Two important knowledge gaps therefore remain. First, the extent to which isolated measures of skeletal muscle loss adequately capture treatment-related physiological vulnerability remains uncertain (10, 11). Second, the relationship between deterioration in physical function and changes in muscle mass is poorly understood, and these processes may not occur in parallel (12, 13). Such heterogeneity may define distinct physiological phenotypes that are differentially associated with treatment tolerance, subsequent clinical outcomes, and survival.

To address these gaps, we constructed a dynamic, data-driven physiological phenotyping framework based on early changes in body composition and physical performance among patients with advanced NSCLC receiving penpulimab-based therapy. We then evaluated the associations of these phenotypes with treatment tolerability and exploratory survival outcomes. Furthermore, we sought to elucidate whether early physiological deterioration might serve as a potential explanatory pathway mediating the association between treatment regimen intensity and subsequent mortality risk after accounting for baseline treatment-selection bias.

By integrating longitudinal physiological assessments with survival analyses, this study seeks to improve the understanding of treatment-related heterogeneity and provide a robust prognostic stratification framework for identifying patients at increased risk of adverse outcomes. Ultimately, preserving physiological resilience may represent an important complementary therapeutic objective alongside tumor control in the management of advanced NSCLC.

## 2 Materials and methods

### 2.1 Study flowchart

**Figure 1:**
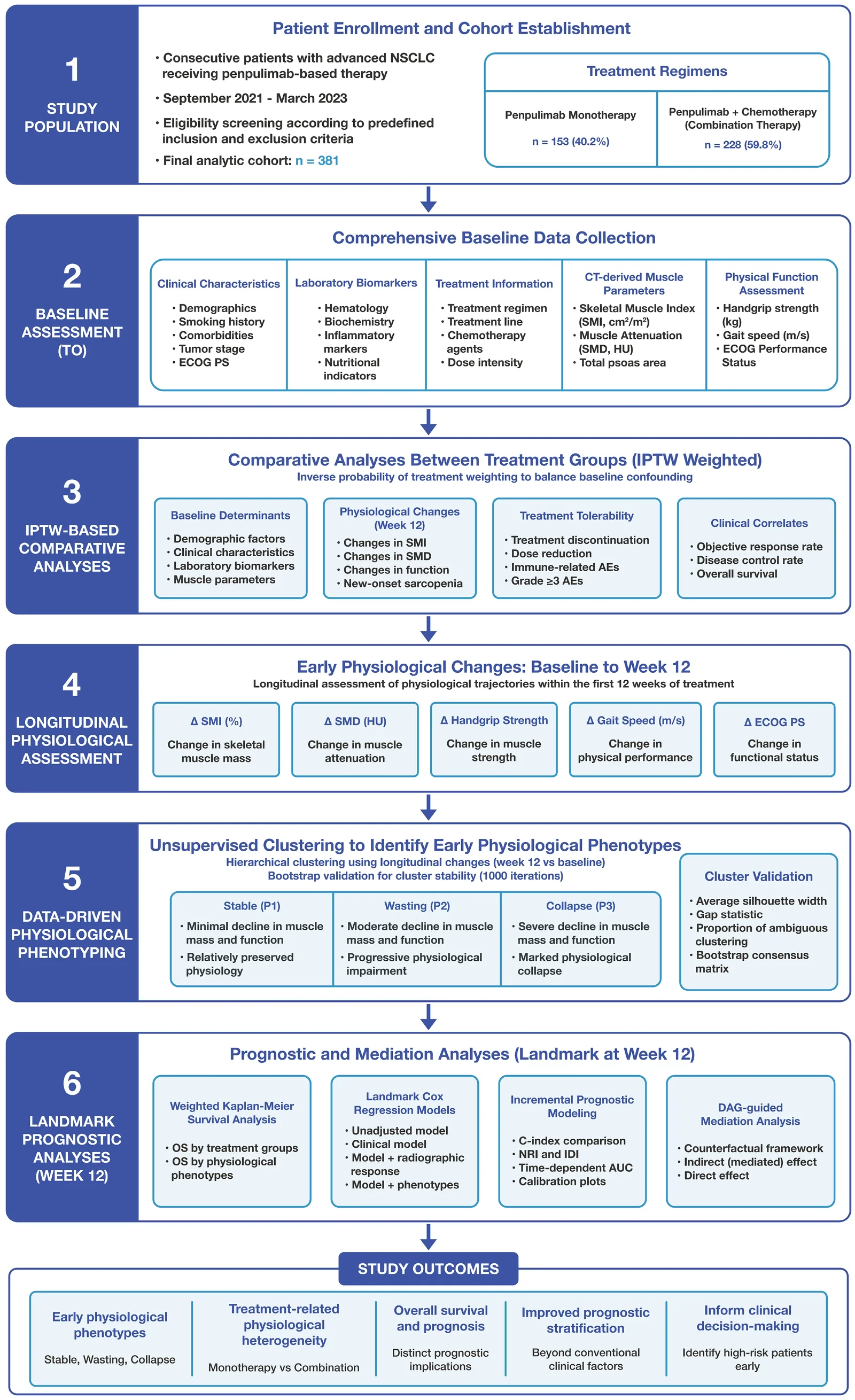
Study design and analytical workflow of dynamic physiological phenotyping in patients with advanced NSCLC receiving penpulimab-based therapy.

This retrospective single-center cohort study was designed to characterize early treatment-related physiological heterogeneity and to explore its clinical implications in patients with advanced NSCLC receiving penpulimab-based therapy. The overall analytical workflow comprised six sequential stages (**Figure 1**).

First, consecutive eligible patients treated with penpulimab between September 2021 and March 2023 were identified according to predefined inclusion and exclusion criteria. Second, comprehensive baseline clinicopathological, laboratory, and treatment-related data were extracted, and paired baseline and 12-week assessments of skeletal muscle parameters and physical function were collected.

Third, early longitudinal physiological changes were quantified using dynamic measures of skeletal muscle mass, muscle quality, and functional performance. Fourth, unsupervised hierarchical clustering was applied to identify data-driven early physiological phenotypes, with internal validation performed using bootstrap resampling.

Fifth, inverse probability of treatment weighting (IPTW) was applied to balance baseline differences between treatment groups, after which treatment-associated physiological changes, baseline determinants, and clinical characteristics of the identified phenotypes were evaluated.

Finally, a prespecified 12-week landmark framework was implemented to minimize immortal time bias, and the association between physiological phenotypes and overall survival (OS) was explored using weighted survival analyses, incremental prognostic modeling, and exploratory pathway analyses. Given the retrospective observational design, survival analyses were considered exploratory and were interpreted cautiously.

### 2.2 Patient population

This single-center retrospective cohort study consecutively identified patients with advanced NSCLC who received penpulimab-based therapy at the Second Affiliated Hospital of Xingtai Medical College between September 2021 and March 2023. Histological or cytological confirmation of NSCLC was required for study inclusion. Follow-up continued the data cutoff date of September 30, 2025.

The study was approved by the Institutional Ethics Committee of the Second Affiliated Hospital of Xingtai Medical College (Approval No. 2025KY031) and was conducted in accordance with the Declaration of Helsinki and its subsequent amendments. Owing to the retrospective design and use of de-identified data, the requirement for written informed consent was waived.

Patients were eligible if they met all of the following criteria:

- Histologically or cytologically confirmed NSCLC;
- Stage IVB disease or unresectable recurrent/metastatic disease according to the eighth edition of the American Joint Committee on Cancer staging system (14);
- Receipt of penpulimab either as monotherapy or in combination with platinum-based chemotherapy as first-line or subsequent-line systemic treatment;
- Completion of at least two treatment cycles and at least one radiological response assessment according to Response Evaluation Criteria in Solid Tumors (RECIST) version 1.1 (15);
- Availability of abdominal or thoracic computed tomography (CT) scans obtained within 30 days before treatment initiation and repeated at approximately 12 weeks (± 2 weeks) after treatment initiation for assessment of skeletal muscle parameters.

Patients were excluded if they had:

- Another active primary malignancy;
- CT images of inadequate quality, incomplete third lumbar vertebral (L3) coverage, or substantial imaging artifacts that prevented reliable skeletal muscle quantification;
- Missing baseline clinical, laboratory, treatment, or radiological data required for the primary analyses;
- Receipt of interventions that might substantially alter body composition during the landmark period, including long-term systemic corticosteroid therapy, parenteral nutritional support, or major surgery.
- Patients harboring actionable driver alterations, including EGFR mutations, ALK rearrangements, ROS1 rearrangements, RET fusions, BRAF V600E mutations, HER2 mutations, MET exon 14 skipping mutations, and NTRK fusions, were excluded prior to cohort assembly.

### 2.3 Treatment protocols

Treatment strategies were determined by a multidisciplinary thoracic oncology team according to contemporary clinical guidelines, tumor characteristics, prior treatment history, programmed death-ligand 1 (PD-L1) expression status, disease burden, performance status, comorbidities, and patient preference. Because this was a real-world retrospective study, no predefined institutional criteria or biomarker thresholds were used to mandate the selection of penpulimab monotherapy versus combination therapy.

All patients received penpulimab at a fixed dose of 200 mg administered intravenously every 3 weeks (Q3W), either as monotherapy or in combination with platinum-based chemotherapy.

Patients in the monotherapy group received penpulimab until radiologically confirmed disease progression, unacceptable toxicity, death, withdrawal of consent, or physician decision.

Patients in the combination group received penpulimab plus platinum-based doublet chemotherapy consisting of nab-paclitaxel (100–125 mg/m² intravenously on days 1, 8, and 15) and cisplatin (75 mg/m² intravenously on day 1) (16, 17). Treatment was administered in 21-day cycles. Chemotherapy doses were determined according to body surface area and could be modified according to age, ECOG performance status, hematologic parameters, hepatic function, and renal function (18).

Tumor response was evaluated approximately every 12 weeks according to RECIST version 1.1 as part of routine clinical practice (15).

Treatment-related adverse events were graded according to the National Cancer Institute Common Terminology Criteria for Adverse Events, version 5.0. For grade ≥3 adverse events, treatment delays, temporary interruptions, or chemotherapy dose reductions were permitted according to institutional practice. Immunotherapy could be resumed after toxicities improved to grade ≤1 following physician assessment (19).

Relative dose intensity (RDI) and permanent treatment discontinuation were recorded and incorporated into subsequent sensitivity analyses.

### 2.4 Clinical and laboratory data collection

Clinical, pathological, treatment-related, and laboratory data were retrospectively extracted from the institutional electronic medical record system and independently verified by two investigators. Baseline variables were defined as measurements obtained within 7 days before initiation of penpulimab therapy. Demographic and clinicopathological characteristics included age, sex, body mass index (BMI), smoking history, histological subtype, PD-L1 tumor proportion score (TPS), AJCC TNM stage (8th edition), metastatic sites, treatment line, and baseline Eastern Cooperative Oncology Group performance status (ECOG PS).

Baseline laboratory parameters included serum albumin, hemoglobin, C-reactive protein (CRP), absolute neutrophil count, absolute lymphocyte count, and platelet count. Inflammation-related indices, including the neutrophil-to-lymphocyte ratio (NLR) and platelet-to-lymphocyte ratio (PLR), were calculated as the absolute neutrophil or platelet count divided by the absolute lymphocyte count. These variables were selected to reflect nutritional status, hematological reserve, and systemic inflammatory activity.

Longitudinal clinical and laboratory measurements during the first 12 weeks after treatment initiation were collected to characterize early treatment-related physiological changes. Serial assessments included body weight, ECOG PS, complete blood counts, and CRP levels. Early inflammatory kinetics were quantified by calculating the change velocity of NLR and CRP between baseline and week 12: slope = (week 12 value − baseline value)/time interval (months). Positive and negative slopes indicated increased and attenuated inflammatory activity, respectively. These dynamic indices were subsequently integrated with physiological parameters for early phenotype classification and outcome analyses.

### 2.5 Assessment of skeletal muscle and physical function

Quantitative assessment of skeletal muscle was performed using pretreatment and 12-week follow-up CT images obtained at the third lumbar vertebral (L3) level, which has been validated as a representative surrogate for whole-body skeletal muscle mass (20). Skeletal muscle area (SMA) was segmented using standardized Hounsfield unit (HU) thresholds ranging from −29 to +150 HU (21). The skeletal muscle index (SMI) was calculated by normalizing SMA to height squared (cm²/m²), and skeletal muscle density (SMD) was defined as the mean attenuation value (HU) of the segmented muscle area, reflecting skeletal muscle quality (22).

Baseline sarcopenia status was determined according to the Asian Working Group for Sarcopenia 2019 (AWGS 2019) criteria using sex-specific SMI thresholds for Asian patients with cancer (< 36.0 cm²/m² for men and < 29.0 cm²/m² for women) (23, 24). Baseline physical function was assessed using handgrip strength, six-meter gait speed, and ECOG PS, which were used to characterize patients’ initial physiological reserve.

To characterize early treatment-related physiological evolution, paired assessments at baseline and the prespecified 12-week landmark were used to calculate longitudinal changes in skeletal muscle and functional parameters. The derived dynamic variables included percentage change in SMI (ΔSMI%), change in SMD (ΔSMD), change in gait speed, change in handgrip strength, and change in ECOG PS. These longitudinal physiological parameters were subsequently integrated for the identification of early treatment-response phenotypes.

### 2.6 Data-driven identification and internal validation of early physiological phenotypes

Because no universally accepted framework currently exists for classifying early treatment-related physiological deterioration during systemic therapy, physiological phenotypes were identified using an exploratory, data-driven approach rather than predefined clinical criteria (25).

Five standardized variables representing 12-week changes in skeletal muscle and physical function (ΔSMI%, ΔSMD, Δgait speed, Δhandgrip strength, and ΔECOG PS) were incorporated into an unsupervised hierarchical clustering analysis. Prior to clustering, all variables were standardized using z-score transformation, and the direction of ECOG PS was reversed to ensure that higher values consistently represented better physiological status. Euclidean distance and Ward’s minimum variance linkage method were applied to generate the clustering structure. The optimal number of clusters was determined based on dendrogram characteristics, cluster separation, and clinical interpretability.

The clustering procedure identified three distinct physiological phenotypes. Patients with relatively preserved skeletal muscle and functional status were classified as P1 (Stable). Patients characterized by predominant skeletal muscle decline with relatively maintained functional capacity were classified as P2 (Wasting). Patients exhibiting concurrent deterioration across both muscular and functional domains were classified as P3 (Collapse). Detailed distributions and characteristics of each phenotype are presented in **Supplementary Table 1**. Phenotype labels were assigned after completion of the clustering procedure and therefore represent descriptive, data-derived classifications rather than prespecified diagnostic categories.

The robustness of the clustering solution was internally evaluated using nonparametric bootstrap resampling with 1,000 iterations. For each bootstrap sample, the complete clustering procedure was repeated, and cluster stability was quantified using mean Jaccard similarity coefficients. A Jaccard coefficient > 0.75 was considered indicative of good cluster reproducibility. Reclassification rates across bootstrap samples were additionally assessed to evaluate assignment consistency (26).

Given the exploratory and unsupervised nature of this approach, these physiological phenotypes should be interpreted as hypothesis-generating classifications requiring validation in independent external cohorts.

### 2.7 Follow-up and endpoint definitions

Patients were followed from the initiation of penpulimab therapy until death, loss to follow-up, or the data cutoff date of September 30, 2025, whichever occurred first. Clinical outcomes were obtained from the institutional electronic medical record system and supplemented by telephone interviews when necessary. The median follow-up duration was estimated using the reverse Kaplan–Meier method.

The primary objective of this study was to identify early treatment-related physiological phenotypes and characterize their clinical determinants during the first 12 weeks of therapy. Overall survival (OS) was evaluated as an exploratory endpoint to assess the prognostic relevance of these phenotypes. OS was defined as the time from the landmark date to death from any cause or the last follow-up.

To minimize potential immortal time bias associated with dynamic longitudinal assessments, a prespecified 12-week landmark analysis was applied. The landmark time point was selected based on completion of early physiological evaluations and the first scheduled radiological response assessment. The landmark cohort was derived from the baseline eligible population according to prespecified criteria. Patients who experienced death, treatment discontinuation due to severe clinical deterioration, or loss to follow-up before the landmark time point were considered unavailable for landmark assessment and were not included in subsequent landmark survival analyses. Therefore, inclusion in landmark survival analyses was strictly conditional on survival to the 12-week landmark date and availability of complete, paired baseline and 12-week physiological assessments. Survival times were calculated from the landmark date rather than the treatment initiation date.

A 12-week landmark was prespecified to evaluate early treatment-related physiological changes during systemic therapy. This time point was selected because it corresponds to approximately 4 treatment cycles of a 21-day regimen, allowing sufficient time for clinically relevant alterations in skeletal muscle and physical function to emerge while preceding the median progression-free survival reported for first-line chemoimmunotherapy in advanced NSCLC. Therefore, the 12-week landmark was not intended to approximate median PFS, but rather to capture early physiological evolution before substantial disease progression-related deterioration occurred.

### 2.8 Data management and quality control

A rigorous data management and quality control protocol was implemented to ensure data integrity, reproducibility, and analytical reliability. An analytical framework was prespecified based on existing clinical evidence and literature consensus before statistical analyses were performed.

All variables required for physiological phenotype classification and exploratory survival analyses, including 12-week CT-derived body composition parameters, functional assessments, and survival outcomes, were systematically defined within this framework. Given the retrospective design of this cohort study, the final sample size was determined by the number of consecutive eligible patients available in the institutional database during the study period, rather than by a prospective sample size calculation.

Patients with missing data in key longitudinal variables required for phenotype assignment were excluded from the primary cluster-based analyses. Missing values among other baseline clinicopathological covariates were rare (< 5%) and were handled using complete-case analysis.

Continuous variables were screened for biologically implausible values, outliers, and potential data-entry errors using graphical inspection and predefined clinical ranges. Suspected discrepancies were cross-verified against the original electronic medical records and source documents. Confirmed transcription errors were corrected, whereas clinically plausible extreme values were retained to preserve the characteristics of the real-world cohort.

All extracted data underwent independent review and verification by two investigators, with discrepancies resolved through consensus. Periodic quality audits were conducted to ensure data completeness and consistency. The final analytical database was locked before statistical analyses, and all downstream analyses were conducted according to the prespecified statistical analysis plan.

### 2.9 Statistical analysis

All statistical analyses were performed according to a prespecified analytical framework. Continuous variables were summarized as mean ± standard deviation (SD) or median (interquartile range [IQR]), and categorical variables were presented as frequencies and percentages. All tests were two-sided, with *P* < 0.05 considered statistically significant. Analyses were performed using R software (version 4.5.2). Continuous variables were compared using weighted or unweighted methods as appropriate, and categorical variables were compared using *χ²* tests, Fisher exact tests, or weighted regression models where applicable.

#### 2.9.1 Propensity score estimation and inverse probability of treatment weighting

To minimize treatment-selection bias and baseline confounding between the penpulimab monotherapy and combination therapy groups, IPTW based on propensity scores was applied.

Propensity scores representing the probability of receiving combination therapy were estimated using a multivariable logistic regression model incorporating prespecified baseline pre-treatment covariates considered clinically relevant to treatment allocation and subsequent outcomes. These variables included age, sex, BMI, smoking history, histological subtype, PD-L1 TPS, ECOG performance status, number of metastatic organs, liver metastasis, brain metastasis, bone metastasis, driver mutation status, albumin concentration, CRP, NLR, SMI, SMD, and baseline sarcopenia status.

Stabilized weights were calculated to reduce the influence of extreme observations while preserving the effective sample structure (27). Covariate balance before and after weighting was assessed using absolute standardized mean differences (ASMDs), with an absolute SMD < 0.10 considered indicative of adequate balance (28). Propensity score overlap, weight distributions, Love plots, and the effective sample size (ESS) were rigorously evaluated to monitor potential sample attrition and pseudo-population inflation; these diagnostic measures are detailed in the **Supplementary Materials** (29).

Sensitivity analyses were conducted using weight truncation (1st–99th and 5th–95th percentiles), overlap weighting, doubly robust estimation, and conventional multivariable regression adjustment (30).

Covariates were selected *a priori* based on clinical knowledge and previous literature and were chosen to represent factors potentially associated with both treatment assignment and survival outcomes.

#### 2.9.2 Baseline determinants and clinical correlates of physiological phenotypes

To identify baseline factors associated with transitions to the data-driven physiological phenotypes, IPTW-weighted multinomial logistic regression models were constructed using the Stable phenotype (P1) as the reference category. Candidate predictors were prespecified according to clinical relevance and included demographic characteristics, tumor burden, inflammatory and nutritional indicators, baseline muscle parameters, and treatment regimen. Results are presented as odds ratios (ORs) and corresponding 95% confidence intervals (CIs).

Associations between physiological phenotypes and treatment-related outcomes, including Grade ≥3 adverse events, hematologic toxicity, immune-related adverse events, permanent treatment discontinuation, objective response rate (ORR), and disease control rate (DCR), were examined using IPTW-weighted logistic regression models. Formal treatment-by-phenotype interaction terms were incorporated to evaluate potential effect modification.

Additional stratified and sensitivity analyses accounting for RECIST-defined tumor response categories, documented infection, systemic corticosteroid use, and baseline cachexia status were performed to examine the robustness of phenotype-associated risks. Given the observational nature of these analyses, the identified associations were interpreted as measures of clinical relevance rather than evidence of causality.

#### 2.9.3 Landmark survival analysis and incremental prognostic modeling

Survival analyses were restricted to patients who remained alive and clinically evaluable at the prespecified 12-week landmark to minimize immortal time bias.

OS was calculated from the landmark onward and estimated using IPTW-weighted Kaplan–Meier methods. Survival distributions were compared using weighted log-rank tests.

To evaluate the incremental prognostic contribution of early physiological phenotypes, three sequential landmark Cox proportional hazards models were constructed:

- Model 1: baseline demographic and clinical variables only (age, sex, BMI, ECOG performance status, treatment regimen, and PD-L1 TPS);
- Model 2: Model 1 plus the early physiological phenotypes (P2 and P3);
- Model 3: Model 2 plus the treatment-by-phenotype interaction term (Combination × P3).

Adjusted hazard ratios (aHRs) and corresponding 95% CIs were reported. Model discrimination and fit were evaluated using Harrell’s concordance index (C-index), changes in C-index (ΔC-index), likelihood ratio tests (LRTs), and Akaike information criterion (AIC). The proportional hazards assumption was assessed using Schoenfeld residuals.

As a sensitivity analysis, the final model was additionally adjusted for RECIST-defined best response, documented infection, systemic corticosteroid use, and baseline cachexia status. To avoid overfitting and ensure model parsimony within this historical cohort, the number of covariates in the multivariable models was constrained based on the events-per-variable (EPV) principle, ensuring approximately 10–15 events per estimated parameter where feasible.

#### 2.9.4 Directed acyclic graph-guided exploratory mediation analysis

**Figure 2:**
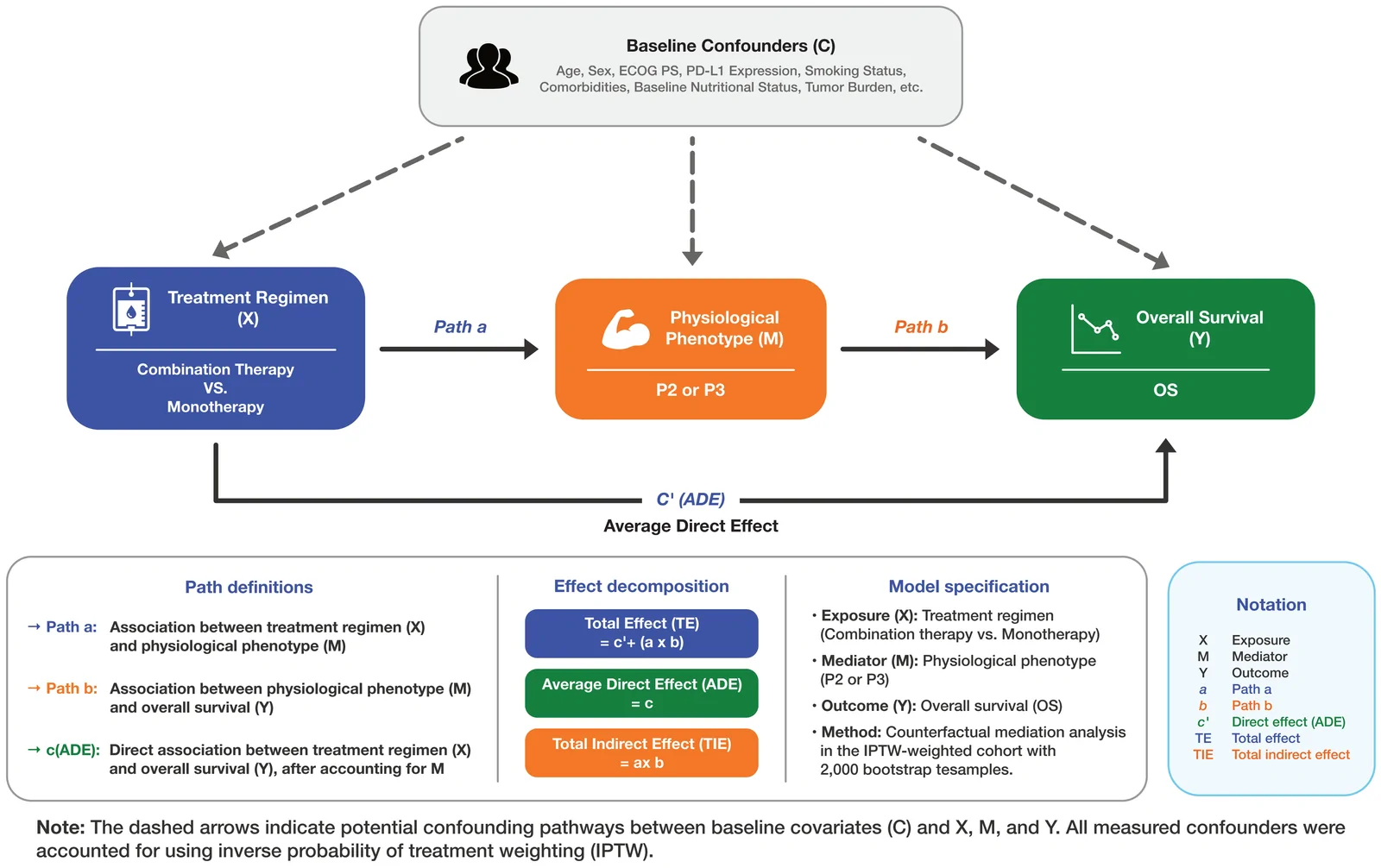
Conceptual framework of the counterfactual mediation analysis evaluating the potential role of early physiological phenotypes in the association between treatment regimen and overall survival. The DAG illustrates the hypothesized relationships among treatment regimen, early physiological phenotypes, and overall survival and provides the conceptual framework for the mediation analyses. Model Components * Exposure (X): Treatment regimen, coded as a binary variable with Combination therapy as the reference category (0) and Monotherapy coded as 1. * Mediator (*M*): Twelve-week physiological phenotype (Wasting [P2] or Collapse [P3]). * Outcome (*Y*): OS. * Baseline Confounders (*C*): Measured baseline characteristics, including age, sex, ECOG performance status, PD-L1 expression, smoking status, comorbidities, baseline nutritional status, and tumor burden, that may influence the associations among treatment regimen, physiological phenotypes, and survival (indicated by dashed arrows). Path Definitions and Effect Decomposition * Path a: Estimated association between treatment regimen (X) and the probability of developing vulnerable physiological phenotypes (M). * Path b: Estimated association between physiological phenotypes (*M*) and overall survival (*Y*), conditional on treatment regimen and measured confounders. * Average Direct Effect (ADE, *c′*): Estimated association between treatment regimen (*X*) and survival (Y) not operating through the specified physiological phenotype. * Total Indirect Effect (TIE, *a × b*): Estimated indirect association potentially operating through treatment-related physiological phenotypes (*M*). * TE: Overall association between treatment regimen and survival, represented as the sum of the direct and indirect effect estimates. Methodological Notes: To reduce measured confounding, baseline covariates were balanced using IPTW. Effect estimates and corresponding 95% confidence intervals were obtained from counterfactual mediation analyses with 2,000 bootstrap resamples in the IPTW-weighted 12-week landmark cohort. Because mediation analyses were performed within an observational framework, the identified pathways should be interpreted as potential explanatory pathways rather than definitive evidence of causality. ADE, Average Direct Effect; CI, Confidence Interval; DAG, Directed Acyclic Graph; ECOG PS, Eastern Cooperative Oncology Group performance status; IPTW, Inverse Probability of Treatment Weighting; OS, Overall Survival; P2, Wasting phenotype; P3, Collapse phenotype; PD-L1, Programmed Death-Ligand 1; TE, Total Effect; TIE, Total Indirect Effect.

A directed acyclic graph (DAG) was constructed *a priori* based on existing biological and clinical evidence to identify a minimally sufficient set of baseline covariates for confounding control and to provide a conceptual framework for mediation analyses (**Figure 2**).

Within the IPTW-weighted landmark cohort, an exploratory counterfactual mediation analysis was performed, specifying treatment regimen (combination therapy versus monotherapy) as the exposure (*X*), the multi-categorical physiological phenotype (P2 and P3, with the Stable phenotype [P1] as the reference) as the mediator (*M*), and OS as the outcome (*Y*).

The total association between treatment regimen and mortality was decomposed into the average direct effect (ADE) and indirect effects operating through the physiological phenotypes. Mediation effects were estimated on the log-hazard ratio scale and point estimates with corresponding 95% CIs were obtained using non-parametric bootstrap resampling with 2,000 iterations.

Because P2 and P3 may share overlapping biological and clinical mechanisms, mediation proportions were interpreted non-additively. E-values were calculated to assess the robustness of the estimated indirect associations to potential unmeasured mediator–outcome confounding. Given the observational design of the present study, these analyses were considered exploratory and hypothesis-generating and should not be interpreted as establishing causal relationships.

## 3 Results

### 3.1 Baseline characteristics and propensity score analyses

A total of 381 eligible patients were included in the final analysis, comprising 153 patients (40.2%) in the Monotherapy group and 228 patients (59.8%) in the Combination group (**Table 1**).

**Table 1:** Baseline demographic and clinical characteristics of the study population according to treatment regimens.

Characteristic	Total (N = 381)	Monotherapy (n=153)	Combination (n=228)	*P _value_*
Demographics				
Age, median (IQR), years	65 (58–71)	66 (59–72)	64 (57–70)	0.124
Sex (Male), n (%)	268 (70.3)	105 (68.6)	163 (71.5)	0.548
BMI, kg/m²	23.41 ± 3.13	23.57 ± 2.98	23.31 ± 3.22	0.432
Smoking status (Ever), n (%)	215 (56.4)	82 (53.6)	133 (58.3)	0.364
Tumor characteristics				
Histology, n (%)				<0.001
Adenocarcinoma	245 (64.3)	118 (77.1)	127 (55.7)	
Squamous cell carcinoma	112 (29.4)	25 (16.3)	87 (38.2)	
Others	24 (6.3)	10 (6.5)	14 (6.1)	
PD-L1 TPS, n (%)				<0.001
<1%	85 (22.3)	0 (0.0)	85 (37.3)	
1–49%	142 (37.3)	32 (20.9)	110 (48.2)	
≥50%	154 (40.4)	121 (79.1)	33 (14.5)	
TNM stage (IVB), n (%)	210 (55.1)	82 (53.6)	128 (56.1)	0.627
Number of metastatic organs, median (IQR)	2 (1–2)	1 (1–2)	2 (1–2)	0.031
Liver metastasis, n (%)	58 (15.2)	18 (11.8)	40 (17.5)	0.128
Bone metastasis, n (%)	92 (24.1)	35 (22.9)	57 (25.0)	0.635
Brain metastasis, n (%)	64 (16.8)	28 (18.3)	36 (15.8)	0.519
Adrenal metastasis, n (%)	41 (10.8)	14 (9.2)	27 (11.8)	0.418
Line of therapy (1L), n (%)	312 (81.9)	128 (83.7)	184 (80.7)	0.463
Functional and muscle status				
ECOG PS (1), n (%)	242 (63.5)	95 (62.1)	147 (64.5)	0.636
SMI, cm²/m²	42.16 ± 6.41	42.48 ± 6.22	41.94 ± 6.54	0.418
Muscle attenuation, HU	34.22 ± 7.85	34.51 ± 7.43	34.02 ± 8.13	0.552
Handgrip strength, kg	28.45 ± 8.21	28.71 ± 7.95	28.28 ± 8.39	0.615
Gait speed, m/s	0.98 ± 0.21	1.00 ± 0.21	0.97 ± 0.22	0.324
Nutritional and inflammatory markers				
Albumin, g/L	38.41 ± 4.22	38.65 ± 4.01	38.25 ± 4.35	0.362
Hemoglobin, g/L	124.51 ± 15.34	125.87 ± 14.85	123.60 ± 15.62	0.158
NLR, median (IQR)	3.41 (2.12–5.13)	3.13 (1.98–4.85)	3.60 (2.22–5.31)	0.042
PLR, median (IQR)	182.4 (142.1–245.3)	175.5 (138.4–232.1)	187.1 (145.2–254.8)	0.085
CRP, median (IQR), mg/L	12.4 (4.2–32.5)	11.3 (3.8–28.7)	13.2 (4.5–35.1)	0.142

Values are presented as median (interquartile range [IQR]), mean ± SD, or number (%), as appropriate. Comparisons between treatment groups were performed using the Mann–Whitney *U* test or Student’s *t*-test for continuous variables, and the Chi-square test or Fisher’s exact test for categorical variables, as appropriate. All enrolled patients underwent molecular testing according to institutional standards. Patients harboring actionable driver alterations, including EGFR mutations, ALK rearrangements, ROS1 rearrangements, RET fusions, BRAF V600E mutations, HER2 mutations, MET exon 14 skipping mutations, and NTRK fusions, were excluded prior to cohort assembly. Number of metastatic organs was used as a surrogate measure of baseline tumor burden.

BMI, body mass index; CRP, C-reactive protein; ECOG PS, Eastern Cooperative Oncology Group performance status; HU, Hounsfield units; IQR, interquartile range; NLR, neutrophil-to-lymphocyte ratio; PD-L1 TPS, programmed death-ligand 1 tumor proportion score; PLR, platelet-to-lymphocyte ratio; SD, standard deviation; SMI, skeletal muscle index; TNM, tumor-node-metastasis; 1L, first-line.

Before weighting, several clinically relevant imbalances were observed between the treatment groups. Patients receiving monotherapy were more likely to have adenocarcinoma and high PD-L1 expression, whereas those receiving combination therapy tended to have squamous cell carcinoma, a greater metastatic burden, and slightly higher baseline inflammatory status, as reflected by the neutrophil-to-lymphocyte ratio (**Table 1**). These disparities were further reflected by substantial standardized mean differences, particularly for PD-L1 TPS (SMD = 1.428) and histological subtype (SMD = 0.458), suggesting the presence of clinically meaningful baseline imbalances and potential treatment-selection bias (**Table 2**).

**Table 2:** Comparison of baseline characteristics between monotherapy and combination groups before and after IPTW.

Characteristic	Monotherapy (n=153)	Combination (n=228)	SMD	Monotherapy (weighted)	Combination (weighted)	SMD
Demographic characteristics						
Age, median (IQR), years	66 (59–72)	64 (57–70)	0.165	65 (58–71)	65 (58–71)	0.012
Male sex, n (%)	111 (72.5)	171 (75.0)	0.057	140.6 (74.6)	143.5 (74.5)	0.003
BMI, mean ± SD, kg/m²	23.1 ± 3.4	22.7 ± 3.6	0.113	22.9 ± 3.5	22.9 ± 3.4	0.008
Smoking history, n (%)	98 (64.1)	160 (70.2)	0.131	128.2 (68.0)	131.0 (68.1)	0.004
Tumor characteristics						
Histology, n (%)						
Adenocarcinoma	118 (77.1)	127 (55.7)	0.458	80.0 (42.4)	81.0 (42.1)	0.007
Squamous cell carcinoma	25 (16.3)	87 (38.2)		108.5 (57.6)	111.5 (57.9)	
Other	10 (6.6)	14 (6.1)	0	0		
PD-L1 TPS, n (%)						
≥50%	121 (79.1)	33 (14.5)	1.428	78.0 (41.4)	79.0 (41.0)	0.009
1–49%	32 (20.9)	110 (48.2)		71.0 (37.7)	73.0 (37.9)	
<1%	0 (0.0)	85 (37.3)		39.5 (20.9)	40.5 (21.1)	
Unknown	0 (0.0)	0 (0.0)	0	0		
ECOG PS = 1, n (%)	95 (62.1)	147 (64.5)	0.049	120.0 (63.7)	123.0 (63.9)	0.005
Number of metastatic organs, median (IQR)	1 (1–2)	2 (1–2)	0.221	1 (1–2)	1 (1–2)	0.032
Liver metastasis, n (%)	24 (15.7)	47 (20.6)	0.128	35.2 (18.7)	36.0 (18.7)	0.002
Brain metastasis, n (%)	18 (11.8)	34 (14.9)	0.092	24.3 (12.9)	24.6 (12.8)	0.004
Bone metastasis, n (%)	41 (26.8)	72 (31.6)	0.106	56.3 (29.9)	57.6 (29.9)	0.002
Driver mutation positive, n (%)	12 (7.8)	19 (8.3)	0.018	15.8 (8.4)	16.2 (8.4)	0.001
Inflammatory and nutritional characteristics						
Albumin, mean ± SD, g/L	39.8 ± 4.3	39.2 ± 4.7	0.133	39.5 ± 4.5	39.4 ± 4.6	0.015
CRP, median (IQR), mg/L	11.3 (3.8–28.7)	13.2 (4.5–35.1)	0.151	12.4 (4.2–32.5)	12.4 (4.1–32.8)	0.011
NLR, median (IQR)	3.13 (2.0–4.9)	3.60 (2.2–5.3)	0.208	3.42 (2.1–5.1)	3.41 (2.1–5.1)	0.004
Muscle-related characteristics						
SMI, mean ± SD, cm²/m²	42.48 ± 6.22	41.94 ± 6.54	0.085	42.12 ± 6.35	42.16 ± 6.42	0.006
SMD, mean ± SD, HU	36.8 ± 8.5	35.9 ± 8.7	0.104	36.3 ± 8.6	36.2 ± 8.5	0.008
Baseline sarcopenia, n (%)	58 (37.9)	95 (41.7)	0.078	75.1 (39.8)	76.5 (39.7)	0.003
ESS	153	228	–	139.8	210.7	–

Values are presented as median (interquartile range [IQR]), mean ± SD, or number (%), as appropriate. IPTW was applied to generate a weighted pseudo-population with balanced baseline characteristics between the monotherapy and combination therapy groups. Covariate balance before and after weighting was assessed using absolute SMDs, with an absolute SMD <0.10 considered indicative of negligible imbalance and adequate covariate balance. ESSs were calculated after weighting to evaluate the amount of statistical information retained in the weighted cohort. Weighted sample sizes represent the sum of stabilized IPTW weights and therefore do not correspond to actual patient counts.

IPTW, inverse probability of treatment weighting; ESS, effective sample size; SMD, standardized mean difference; IQR, interquartile range; BMI, body mass index; PD-L1 TPS, programmed death-ligand 1 tumor proportion score; ECOG PS, Eastern Cooperative Oncology Group performance status; SMI, skeletal muscle index; HU, Hounsfield units; CRP, C-reactive protein; NLR, neutrophil-to-lymphocyte ratio; PLR, platelet-to-lymphocyte ratio.

To address these imbalances, IPTW was applied. Propensity score distributions demonstrated adequate overlap between the treatment groups, suggesting sufficient common support for weighted analyses (**Supplementary Figure 1B**). After weighting, all baseline covariates achieved satisfactory balance, with all absolute ASMDs falling below the prespecified threshold of 0.10 (**Table 2**, **Supplementary Figures 1A, C**). The effective sample size remained largely preserved (Monotherapy: 148.9 vs. 153; Combination: 211.2 vs. 228), suggesting limited information loss following weighting (**Supplementary Figure 1D**). Sensitivity and alternative weighting analyses—including weight trimming, overlap weighting, doubly robust estimation, and multivariable Cox models—yielded broadly similar estimates (**Supplementary Figures 1E, F**), providing reassurance regarding the robustness of the primary analyses, although residual confounding due to unmeasured factors cannot be completely excluded. Furthermore, the ESS remained sufficiently large post-weighting, indicating that optimal covariate balance was achieved without a substantial loss of statistical efficiency (**Table 2**).

### 3.2 Early physiological changes, treatment tolerability, and PD-L1-stratified sensitivity analyses

At the prespecified 12-week landmark (T1), patients receiving combination therapy exhibited a less favorable physiological profile than those receiving monotherapy (**Table 3**). Compared with the Monotherapy group, the Combination group had a higher incidence of new-onset functional impairment (21.8% vs. 14.9%, *P* = 0.028), greater declines in physical performance measures, including handgrip strength and gait speed (both *P* < 0.001), and more pronounced muscle deterioration, as reflected by larger reductions in skeletal muscle index and muscle attenuation and a higher incidence of new-onset sarcopenia (16.1% vs. 8.0%, *P* = 0.018).

**Table 3:** Early physiological changes and treatment tolerability at the 12-week landmark according to treatment regimens in the IPTW-weighted cohort.

Category/outcome	Weighted total (N = 381)	Monotherapy (n = 188.5)*	Combination (n = 192.5)*	*P _value_*
Functional decline				
New-onset functional impairment (ECOG PS ≥2), n (%)	70 (18.4%)	28 (14.9%)	42 (21.8%)	0.028
Change in handgrip strength (%), T1 vs T0	−4.18 ± 2.05	−2.75 ± 1.72	−5.58 ± 2.21	<0.001
Change in 6-m gait speed (%), T1 vs T0	−3.45 ± 1.72	−2.05 ± 1.35	−4.82 ± 1.95	<0.001
Imaging-based muscle changes				
Change in SMI (%), T1 vs T0	−5.02 ± 2.31	−3.12 ± 1.88	−6.88 ± 2.54	<0.001
Change in muscle attenuation (HU), T1 vs T0	−3.15 ± 1.35	−1.82 ± 1.05	−4.45 ± 1.48	<0.001
New-onset sarcopenia, n (%)	46 (12.1%)	15 (8.0%)	31 (16.1%)	0.018
Treatment tolerability and nutritional changes				
Body weight loss (%)	3.35 ± 1.15	2.05 ± 0.92	4.62 ± 1.28	<0.001
RDI (%)	92.5 ± 6.2	96.8 ± 3.1	88.3 ± 7.5	<0.001
Grade ≥3 adverse events, n (%)	115 (30.2%)	34 (18.0%)	81 (42.1%)	<0.001
Inflammatory changes				
Change in NLR, T1 vs T0	0.40 ± 0.11	0.20 ± 0.07	0.60 ± 0.13	<0.001
Change in CRP (mg/L), T1 vs T0	1.82 ± 0.51	1.10 ± 0.28	2.52 ± 0.61	<0.001

Values are presented as weighted mean ± SD or weighted number (%). Comparisons between treatment groups were performed in the IPTW-weighted pseudo-population using weighted linear regression models for continuous variables and weighted logistic regression models for categorical variables. T0 denotes baseline and T1 denotes the prespecified 12-week landmark assessment. Weighted sample sizes represent pseudo-population estimates and therefore may not correspond to integer patient counts. The ESS after weighting was 148.2 for the Monotherapy group and 210.7 for the Combination group, indicating minimal information loss associated with the weighting procedure.

AE, adverse event; CRP, C-reactive protein; ECOG PS, Eastern Cooperative Oncology Group performance status; HU, Hounsfield units; IPTW, inverse probability of treatment weighting; NLR, neutrophil-to-lymphocyte ratio; RDI, relative dose intensity; SD, standard deviation; SMI, skeletal muscle index; ESS, effective sample size.

Combination therapy was additionally associated with greater increases in inflammatory markers (NLR and CRP; both *P* < 0.001) and poorer treatment tolerability, characterized by greater body weight loss, lower relative dose intensity, and a higher incidence of Grade ≥ 3 adverse events (all *P* < 0.001).

To evaluate whether these associations differed according to baseline PD-L1 expression, exploratory sensitivity analyses were performed across the IPTW-weighted TPS ≥ 50%, TPS 1% – 49%, and TPS <1% subgroups (**Table 4**). No significant treatment-by-PD-L1 interactions were observed for any evaluated outcome (all *P_interaction_* > 0.05), and the direction of the associations appeared generally consistent across PD-L1 subgroups.

**Table 4:** Early physiological changes and treatment tolerability at the 12-week landmark according to pd-l1 tumor proportion score subgroups in the IPTW-weighted cohort.

Category/outcome	PD-L1 TPS ≥50% (Mono: 78.0/Combo: 79.0)	PD-L1 TPS 1–49% (Mono: 71.0/Combo: 73.0)	PD-L1 TPS <1%† (Mono: 39.5/Combo: 40.5)	*P_interaction_*
New-onset functional impairment (ECOG PS ≥2), n (%)				
Monotherapy	11 (14.1%)	10 (14.1%)	6 (15.2%)	
Combination	17 (21.5%)	16 (21.9%)	9 (22.2%)	0.87
Change in handgrip strength (%), T1 vs T0				
Monotherapy	−2.72 ± 1.70	−2.78 ± 1.76	−2.80 ± 1.68	
Combination	−5.50 ± 2.18	−5.63 ± 2.23	−5.61 ± 2.19	0.91
Change in 6-m gait speed (%), T1 vs T0				
Monotherapy	−2.02 ± 1.32	−2.09 ± 1.38	−2.05 ± 1.35	
Combination	−4.76 ± 1.91	−4.88 ± 1.98	−4.83 ± 1.94	0.89
Imaging-based muscle changes				
Change in SMI (%), T1 vs T0				
Monotherapy	−3.05 ± 1.85	−3.18 ± 1.92	−3.14 ± 1.90	
Combination	−6.80 ± 2.47	−6.95 ± 2.58	−6.89 ± 2.54	0.84
Change in muscle attenuation (HU), T1 vs T0				
Monotherapy	−1.79 ± 1.04	−1.85 ± 1.07	−1.82 ± 1.05	
Combination	−4.39 ± 1.45	−4.50 ± 1.52	−4.46 ± 1.48	0.92
New-onset sarcopenia, n (%)				
Monotherapy	6 (7.7%)	6 (8.5%)	3 (7.6%)	
Combination	12 (15.2%)	12 (16.4%)	7 (17.3%)	0.81
Treatment tolerability and nutritional changes				
Body weight loss (%)				
Monotherapy	2.03 ± 0.90	2.07 ± 0.94	2.05 ± 0.91	
Combination	4.55 ± 1.24	4.70 ± 1.31	4.60 ± 1.27	0.88
RDI (%)				
Monotherapy	96.9 ± 3.0	96.7 ± 3.2	96.8 ± 3.1	
Combination	88.5 ± 7.4	88.1 ± 7.7	88.4 ± 7.5	0.94
Grade ≥3 adverse events, n (%)				
Monotherapy	14 (17.9%)	13 (18.3%)	7 (17.7%)	
Combination	33 (41.8%)	31 (42.5%)	17 (42.0%)	0.96
Inflammatory changes				
Change in NLR, T1 vs T0				
Monotherapy	0.19 ± 0.07	0.20 ± 0.08	0.20 ± 0.07	
Combination	0.59 ± 0.13	0.61 ± 0.14	0.60 ± 0.13	0.93
Change in CRP (mg/L), T1 vs T0				
Monotherapy	1.08 ± 0.27	1.12 ± 0.29	1.10 ± 0.28	
Combination	2.48 ± 0.59	2.56 ± 0.63	2.53 ± 0.61	0.9

Values are presented as weighted mean ± SD or weighted number (%). Analyses were performed within subgroups defined according to PD-L1 TPS, categorized as <1%, 1–49%, and ≥50%. Weighted subgroup sample sizes represent pseudo-population estimates derived from IPTW and therefore may not correspond to actual patient counts. Interaction P values were obtained from weighted regression models including treatment-by-PD-L1 subgroup interaction terms. No statistically significant treatment-by-PD-L1 interactions were observed for any early physiological, muscular, inflammatory, or tolerability outcome (all *P_interaction_* > 0.05), suggesting that the associations between treatment regimen and early physiological deterioration were generally consistent across PD-L1 expression strata.

**†** Estimates in the PD-L1 TPS <1% subgroup were derived entirely from the IPTW-weighted pseudo-population because no patients with TPS <1% received monotherapy in the original cohort. Therefore, findings in this subgroup should be interpreted cautiously owing to limited empirical support and potential violation of the positivity assumption.

**‡** PD-L1 expression data were available for all included patients; therefore, no patients were classified as having unknown PD-L1 status.

The consistency of findings across PD-L1 subgroups suggests that the observed physiological deterioration associated with combination therapy was unlikely to be explained solely by differences in baseline PD-L1 expression.

CRP, C-reactive protein; ECOG PS, Eastern Cooperative Oncology Group performance status; HU, Hounsfield units; IPTW, inverse probability of treatment weighting; NLR, neutrophil-to-lymphocyte ratio; RDI, relative dose intensity; SD, standard deviation; SMI, skeletal muscle index; TPS, tumor proportion score.

Because no patients with TPS < 1% received monotherapy in the original cohort, findings within this subgroup should be interpreted cautiously because of the limited overlap between treatment groups. Nevertheless, the observed estimates in this subgroup were directionally similar to those observed in the higher PD-L1 expression categories.

### 3.3 Data-driven physiological phenotypes, internal validation, and baseline determinants

To characterize heterogeneity in early treatment-related physiological changes, unsupervised hierarchical clustering of five standardized 12-week change variables (ΔSMI, ΔHU, Δgait speed, Δhandgrip strength, and ΔECOG PS) identified an optimal three-cluster solution (K = 3; silhouette width = 0.58; Jaccard coefficient = 0.84), yielding three distinct physiological phenotypes (**Supplementary Figure 2**; **Supplementary Table 2**).

**Figure 3:**
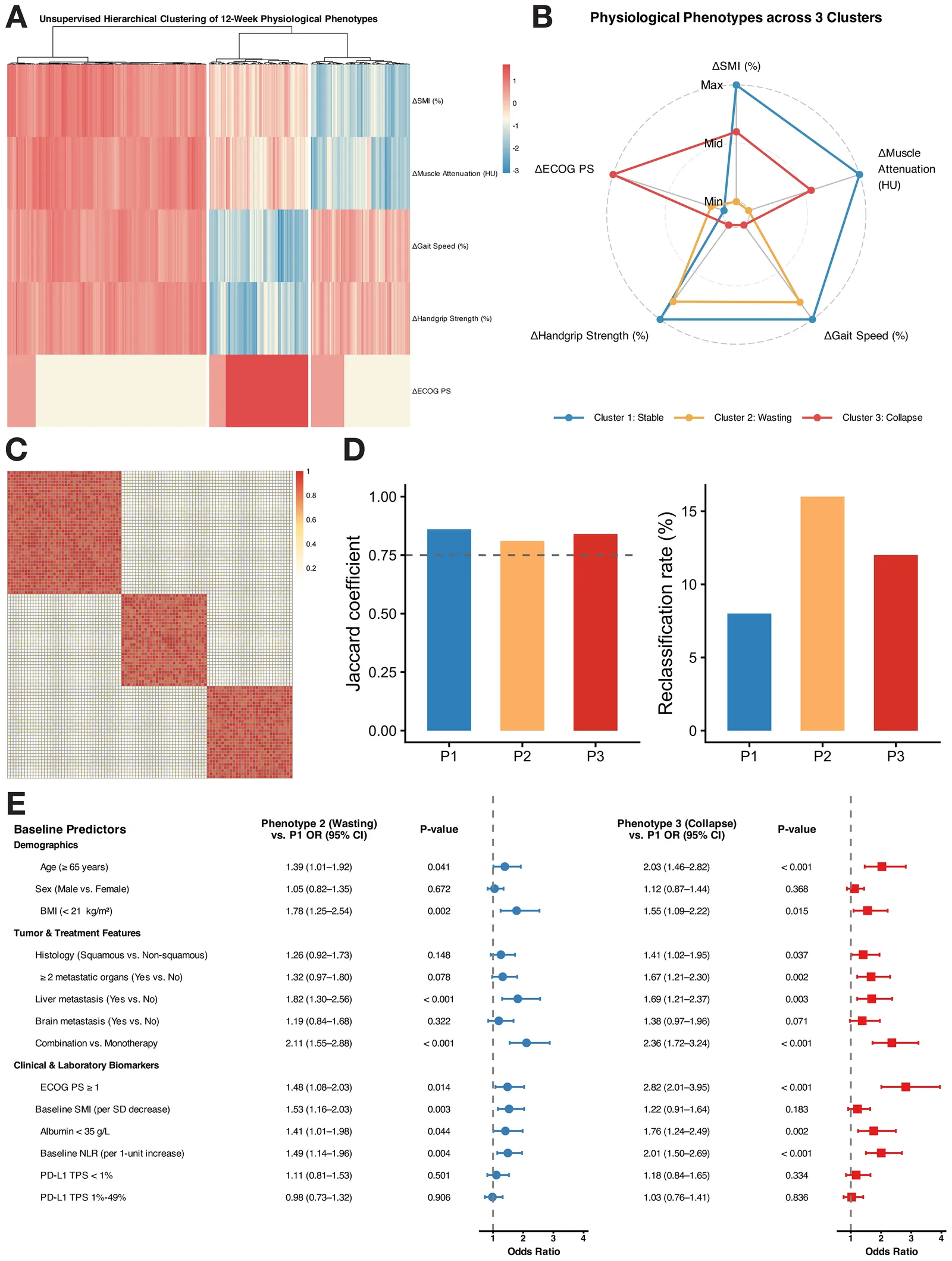
Identification, internal validation, and baseline determinants of data-driven physiological phenotypes at the 12-week landmark. **(A)** Hierarchical clustering heatmap based on Euclidean distance and Ward’s minimum variance method, identifying three distinct physiological phenotypes according to standardized 12-week changes (Δ) in muscular and functional parameters. **(B)** Radar plot illustrating the average physiological profiles of the three identified phenotypes: P1 (Stable, blue), P2 (Wasting, orange), and P3 (Collapse, red). **(C)** Consensus clustering matrix demonstrating clear separation and high assignment consistency among the three phenotypes during iterative resampling. **(D)** Internal validation of cluster stability based on 1,000 bootstrap resamples. Left panel: mean Jaccard similarity coefficients for each phenotype, with the dashed line indicating the prespecified stability threshold of 0.75. Right panel: cluster reclassification rates across bootstrap iterations. **(E)** Forest plot of IPTW-weighted multivariable multinomial logistic regression analyses showing baseline factors associated with transitions to Phenotype 2 (Wasting) and Phenotype 3 (Collapse), using Phenotype 1 (Stable) as the reference category. Estimates are presented as odds ratios (ORs) and 95% confidence intervals (CIs). BMI, body mass index; CI, confidence interval; ECOG PS, Eastern Cooperative Oncology Group performance status; IPTW, inverse probability of treatment weighting; NLR, neutrophil-to-lymphocyte ratio; OR, odds ratio; SMI, skeletal muscle index; TPS, tumor proportion score.

Three distinct physiological phenotypes were identified (**Figures 3A, B**):

- P1 (Stable, *n* = 198.5): minimal physiological deterioration;
- P2 (Wasting, *n* = 98.3): selective muscle wasting with relatively preserved physical function;
- P3 (Collapse, *n* = 84.2): severe functional impairment characterized by marked declines in physical performance and worsening ECOG PS.

Internal validation supported the reproducibility of the clustering solution. Consensus clustering demonstrated clear separation between phenotypes, and bootstrap resampling (1,000 iterations) showed good stability, with all mean Jaccard coefficients exceeding the prespecified threshold of 0.75 and low reclassification rates (**Figures 3C, D**).

Using P1 as the reference category, IPTW-weighted multinomial logistic regression identified distinct baseline predictors for phenotype transitions (**Figure 3E**). Transition to P2 was primarily associated with lower baseline nutritional and muscle reserves, liver metastasis, elevated baseline inflammation (NLR), and combination therapy. In contrast, transition to P3 was associated with older age, impaired baseline performance status, squamous histology, greater metastatic burden (including liver involvement), lower albumin concentration, elevated NLR, and combination therapy. Baseline PD-L1 expression was not significantly associated with either phenotype transition (all *P* > 0.05).

Overall, these findings suggest that early physiological deterioration following treatment is heterogeneous and can be summarized into reproducible data-driven phenotypes. Given the exploratory and unsupervised nature of this analysis, these phenotypes should be regarded as hypothesis-generating classifications pending external validation in independent cohorts.

### 3.4 Clinical relevance of early physiological phenotypes beyond tumor response

Early physiological phenotypes showed significant interactions with treatment regimen in relation to treatment tolerability and short-term antitumor activity (**Supplementary Table 3**). Across both treatment groups, patients classified as vulnerable phenotypes (P2/P3) experienced higher rates of Grade ≥ 3 AEs and permanent treatment discontinuation than those with the Stable phenotype (P1). In the Combination group, the incidence of Grade ≥ 3 AEs increased from 18.2% in P1 to 45.9% in P2/P3 (*P_interaction_* = 0.002), accompanied by lower ORR and DCR.

To assess whether these associations were influenced by tumor response status, analyses were further stratified according to RECIST-defined response categories (**Supplementary Table 4**). The associations between vulnerable phenotypes and adverse clinical outcomes were generally consistent across the CR/PR, SD, and PD subgroups, and no significant phenotype-by-RECIST interactions were observed for any outcome (all *P_interaction_* > 0.05).

Additional adjustment for tumor response status, documented infection, systemic corticosteroid use, and baseline cachexia yielded similar estimates (**Supplementary Table 5**). The associations between vulnerable phenotypes and persistent muscle atrophy, incident cachexia, Grade ≥ 3 AEs, and permanent treatment discontinuation remained largely unchanged after sequential adjustment.

Collectively, these findings suggest that the identified early physiological phenotypes are associated with clinically relevant differences in treatment tolerance and adverse outcomes beyond RECIST-defined tumor response and are unlikely to simply reflect disease progression alone. The proposed physiological phenotyping framework may therefore provide complementary information to conventional tumor response assessment and may help identify patients at increased risk of subsequent adverse clinical outcomes.

### 3.5 Population survival disparities, incremental model performance, and sensitivity analysis

**Figure 4:**
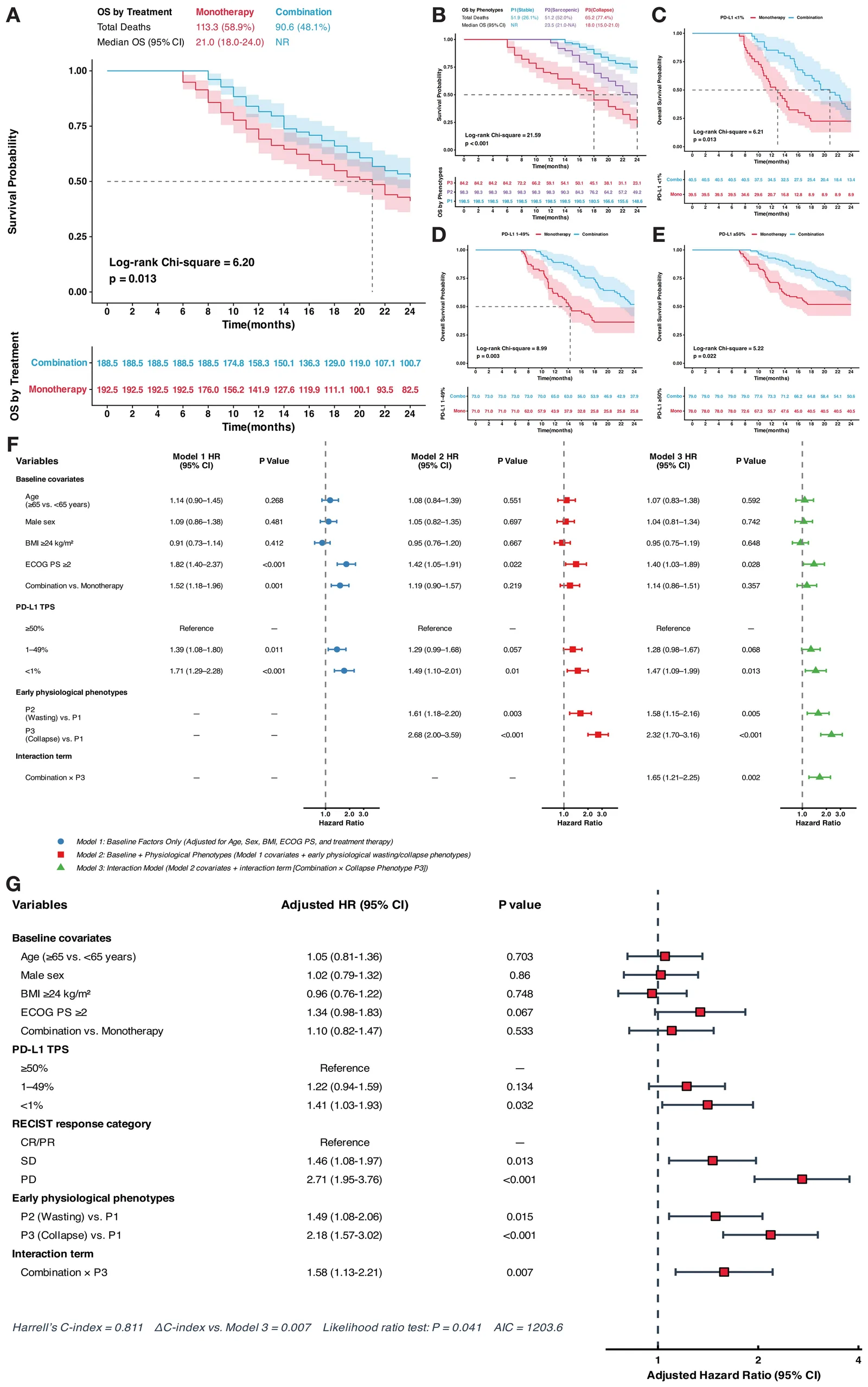
IPTW-weighted overall survival according to treatment regimen and early physiological phenotypes, with sequential prognostic modeling and sensitivity analyses. **(A)** Kaplan–Meier curves for OS according to treatment regimen (Combination therapy vs. Monotherapy) from the 12-week landmark. **(B)** Kaplan–Meier curves for OS according to data-driven physiological phenotypes: Stable (P1), Wasting (P2), and Collapse (P3). **(C–E)** Kaplan–Meier curves comparing combination therapy and monotherapy within baseline PD-L1 TPS subgroups: **(C)** PD-L1 <1%, **(D)** PD-L1 1 – 49%, and **(E)** PD-L1 ≥50%. Shaded areas indicate 95% confidence intervals, and numbers at risk are shown below each panel. **(F)** Forest plots of sequential landmark Cox proportional hazards models evaluating the incremental prognostic value of early physiological phenotypes: * Model 1: Baseline clinical covariates only (age, sex, BMI, ECOG performance status, treatment regimen, and PD-L1 TPS); * Model 2: Model 1 plus physiological phenotypes (P2 and P3); * Model 3: Model 2 plus the treatment-by-phenotype interaction term (Combination × P3). **(G)** Forest plot of the fully adjusted sensitivity model, additionally controlling for RECIST-defined best tumor response (CR/PR, SD, and PD), to assess whether the prognostic associations of physiological phenotypes were independent of radiographic response. All analyses were restricted to patients who survived and remained evaluable at the 12-week landmark to minimize immortal time bias. Harrell’s C-index, ΔC-index, LRT P values, and AIC are displayed to compare model performance. AIC, Akaike information criterion; BMI, body mass index; CI, confidence interval; CR, complete response; ECOG PS, Eastern Cooperative Oncology Group performance status; HR, hazard ratio; LRT, likelihood ratio test; NR, not reached; OS, overall survival; PD, progressive disease; PD-L1 TPS, programmed death-ligand 1 tumor proportion score; PR, partial response; SD, stable disease.

In the IPTW-weighted 12-week landmark pseudo-population, Kaplan–Meier analysis demonstrated a significant OS advantage for patients receiving combination therapy compared with monotherapy (log-rank *χ²* = 6.20, *P* = 0.013; **Figure 4A**). The median OS for the monotherapy group was 21.0 months (95% CI: 18.0 – 24.0), whereas the median OS for the combination therapy group was not reached. The reported numbers of deaths and sample sizes represent IPTW-weighted estimates rather than actual counts from the original cohort.

Survival differed substantially across the data-driven physiological phenotypes (log-rank *P* < 0.001; **Figure 4B**). Patients with the Stable phenotype (P1) had the most favorable prognosis (median OS, not reached), whereas median OS declined to 23.5 months (95% CI: 21.0 – NA) for the Wasting phenotype (P2) and to 18.0 months (95% CI: 15.0 – 21.0) for the Collapse phenotype (P3). Similar patterns were observed in the PD-L1 subgroup analyses (**Figures 4C–E**).

To assess the incremental prognostic value of these phenotypes, three sequential landmark Cox models were constructed in the IPTW-weighted pseudo-population (**Figure 4F**; **Supplementary Table 6**). Model 1, adjusted for baseline covariates, demonstrated moderate discrimination (C-index = 0.654; AIC = 1268.4), and treatment regimen remained independently associated with overall survival. Incorporation of the physiological phenotypes (Model 2) substantially improved model performance (C-index = 0.782; AIC = 1214.8; likelihood ratio test [LRT], *P* < 0.001 vs. Model 1), while the association between treatment regimen and survival was attenuated. Both P2 (HR = 1.61, *P* = 0.003) and P3 (HR = 2.68, *P* < 0.001) remained independent predictors of mortality.

Adding the interaction term between treatment regimen and the Collapse phenotype (Combination × P3) further improved model discrimination (Model 3: C-index = 0.804; AIC = 1207.2; LRT, P = 0.004 vs. Model 2). The interaction term remained significant (HR = 1.65, 95% CI: 1.21 – 2.25, *P* = 0.002), suggesting that the adverse prognostic impact of the Collapse phenotype was more pronounced among patients receiving combination therapy.

To determine whether these findings were independent of radiographic response, an additional model incorporating RECIST-defined responses was constructed (**Figure 4G**). After adjustment for RECIST categories, both P2 (aHR = 1.49, 95% CI: 1.08 – 2.06, *P* = 0.015) and P3 (aHR = 2.18, 95% CI: 1.57 – 3.02, *P* < 0.001) remained independently associated with mortality, and the treatment-by-P3 interaction remained significant (HR = 1.58, 95% CI: 1.13 – 2.21, *P* = 0.007). This fully adjusted model achieved the highest discrimination (C-index = 0.811; AIC = 1203.6; ΔC-index = 0.007 vs. Model 3; LRT, *P* = 0.041).

Collectively, these findings indicate that dynamic physiological phenotypes provide substantial incremental prognostic information beyond conventional clinical variables and radiographic response assessments and identify a subgroup of patients at particularly high risk of adverse outcomes.

### 3.6 Exploratory mediation analyses of early physiological deterioration

To explore potential pathways underlying the observed survival differences, we performed exploratory counterfactual mediation analyses to examine whether early physiological deterioration may partially explain the association between treatment regimen and mortality (**Figure 2**). For these analyses, treatment regimen was coded as a binary exposure variable, with combination therapy serving as the reference category (0) and monotherapy coded as 1. Therefore, positive effect estimates indicate a higher mortality risk associated with monotherapy relative to combination therapy. The total association between treatment regimen and mortality corresponded to a Log-HR of 0.485 (95% CI: 0.124 – 0.846, *P* = 0.012), with an average direct effect of 0.321 (95% CI: 0.015 – 0.627, *P* = 0.045). The combined indirect association through P2 and P3 corresponded to a Log-HR of 0.396 (95% CI: 0.176 – 0.616, *P* < 0.001) (**Supplementary Tables 7**, **S8**).

Both physiological phenotypes demonstrated statistically significant indirect associations with mortality. The P2 (Wasting) pathway showed an indirect association of 0.164 (95% CI: 0.052–0.276, *P* = 0.002), whereas the P3 (Collapse) pathway demonstrated a larger indirect association of 0.232 (95% CI: 0.094 –0.370, *P* < 0.001). Sensitivity analyses yielded similar findings, and the E-value for the P3 pathway (2.53; lower limit, 1.82) suggested moderate robustness to unmeasured confounding (**Supplementary Table 7**).

Overall, these findings suggest that early physiological deterioration may partially explain the association between treatment intensification and subsequent mortality. However, these analyses are exploratory and hypothesis-generating and should not be interpreted as definitive evidence of causal mediation.

### 3.7 Overview of the main findings

The present analyses revealed a coherent pattern of associations. Intensified combination therapy was associated with greater early physiological deterioration, including declines in muscle mass, physical function, and treatment tolerability. These heterogeneous changes could be summarized into three reproducible data-driven phenotypes (Stable, Wasting, and Collapse), which exhibited distinct baseline determinants and were independently associated with subsequent survival outcomes. The identified physiological phenotypes provided prognostic information beyond conventional RECIST-defined tumor response and substantially improved risk discrimination in landmark survival models. Finally, counterfactual mediation analyses suggested that early treatment-induced physiological attrition, particularly the functional decline phenotype (P3), may represent an important potential explanatory pathway linking treatment intensification to subsequent mortality risk. Overall, these findings suggest that longitudinal physiological phenotyping may provide complementary information for understanding treatment heterogeneity and identifying patient subgroups at increased risk of adverse outcomes in advanced NSCLC.

## 4 Discussion

By applying a dynamic, data-driven physiological phenotyping framework within a 12-week landmark design, this study characterized the early physiological trajectories of patients with advanced NSCLC undergoing systemic treatment. We found that, although combination therapy was associated with improved overall survival, it also resulted in substantially greater physiological deterioration, including declines in muscle mass, physical function, and treatment tolerability. Importantly, three reproducible physiological phenotypes—Stable (P1), Wasting (P2), and Collapse (P3)—captured the heterogeneity of these early treatment-related changes and provided prognostic information beyond conventional radiographic response assessments.

In addition, our counterfactual mediation analyses suggested that early treatment-induced physiological attrition may partially explain the association between treatment intensification and subsequent mortality risk. Among the identified trajectories, the functional decline phenotype (P3) showed the strongest estimated indirect association with survival, underscoring the potential importance of preserving physiological resilience during systemic therapy. Collectively, these findings provide a clinical-physiological perspective for understanding the trade-off between therapeutic efficacy and treatment-related physiological deterioration in patients with advanced NSCLC.

### 4.1 The physiological cost and the survival trade-off of treatment intensification

Immuno-chemotherapy has become the standard first-line treatment for advanced NSCLC and has substantially improved outcomes in many patients. However, our findings suggest that these therapeutic gains may come at the expense of considerable physiological deterioration. Compared with monotherapy, combination therapy was associated with greater declines in muscle mass and physical function, a higher incidence of new-onset sarcopenia, and poorer treatment tolerability during the first 12 weeks of treatment.

Several biological mechanisms may contribute to these observations. Cytotoxic chemotherapy can induce systemic inflammation, oxidative stress, and metabolic disturbances that promote skeletal muscle catabolism, whereas immune checkpoint inhibitors may further augment inflammatory and metabolic stress through sustained immune activation (31–33). The cumulative effects of these processes may accelerate physiological depletion in susceptible individuals.

More importantly, our analyses revealed a clinically relevant survival trade-off. Although combination therapy was associated with more favorable survival outcomes among patients who maintained physiological stability (P1), this apparent benefit was substantially attenuated among patients who developed the Wasting (P2) or Collapse (P3) phenotypes. These findings suggest that the net benefit of treatment intensification may depend not only on antitumor efficacy but also on an individual’s capacity to withstand treatment-related physiological stress.

From a clinical perspective, these observations support the concept that physiological resilience represents an important, yet frequently overlooked, determinant of therapeutic benefit in advanced NSCLC. Accordingly, incorporating longitudinal physiological assessments into treatment monitoring may help identify patients who are unlikely to tolerate treatment intensification and who may benefit from early supportive interventions or individualized treatment adaptation strategies.

### 4.2 Functional collapse: dissociation between physiological function and muscle mass

A major contribution of this study is the identification of the functional collapse phenotype (P3). Unlike traditional cachexia frameworks that primarily emphasize muscle mass depletion, P3 was characterized by profound declines in physical function and performance status that appeared disproportionate to the degree of skeletal muscle loss. Although patients with P3 exhibited less severe reductions in skeletal muscle index than those with the Wasting phenotype (P2), they experienced the poorest survival outcomes.

This apparent dissociation between functional performance and muscle quantity suggests that physical function may capture broader dimensions of systemic vulnerability beyond muscle mass alone (34). Patients classified as P3 exhibited the most adverse inflammatory profiles, including markedly elevated NLR and CRP levels, raising the possibility that excessive systemic inflammation contributes to metabolic dysfunction, impaired energy utilization, and deterioration in neuromuscular performance (35, 36). Consequently, functional decline may occur before substantial structural muscle loss becomes clinically apparent.

The early and pronounced separation of survival curves observed in the P3 phenotype further underscores the clinical importance of functional deterioration. Collectively, these findings suggest that assessments of physical performance provide prognostic information that complements conventional measures of body composition and radiographic response. Early identification of patients transitioning toward functional collapse may therefore facilitate timely supportive interventions and more individualized treatment strategies aimed at preserving physiological resilience.

### 4.3 Clinical implications: toward dynamic physiological monitoring and precision supportive care

The present findings have several important clinical implications. First, they support the incorporation of longitudinal physiological monitoring into routine oncological practice. Traditional treatment evaluation in advanced NSCLC relies heavily on radiographic response and treatment-related adverse events; however, our findings suggest that dynamic changes in physical performance and physiological reserve provide additional prognostic information beyond conventional tumor assessments. Serial evaluations of gait speed, muscle strength, body composition, and performance status may help identify patients entering trajectories of accelerated physiological decline and who may benefit from early supportive interventions.

Importantly, despite the availability of consensus definitions and diagnostic algorithms, sarcopenia remains substantially underrecognized and undertreated in routine clinical practice, highlighting the need for pragmatic approaches that facilitate physiological assessment and early intervention (37). The proposed physiological phenotypes are not intended to replace established definitions such as EWGSOP2 or AWGS 2019; rather, they complement existing frameworks by capturing dynamic treatment-related changes in muscle and physical function. Because these phenotypes rely largely on measurements already routinely obtained in oncology practice, including CT imaging and functional assessments, they may represent a pragmatic approach for integrating physiological monitoring into routine cancer care. The ability to distinguish patients with predominant muscle wasting from those experiencing global functional collapse may also provide additional information for risk stratification beyond conventional sarcopenia categories. Emerging evidence further suggests that respiratory sarcopenia may represent an additional dimension of physiological vulnerability in patients with NSCLC, and future studies incorporating respiratory muscle assessments may help further refine dynamic physiological phenotyping (38).

Second, the observed heterogeneity in physiological trajectories suggests that treatment intensification should not be evaluated solely on the basis of antitumor efficacy. The attenuation of survival benefit among patients who developed the Wasting (P2) and Collapse (P3) phenotypes indicates that physiological resilience may be an important determinant of treatment tolerance and net clinical benefit. Early physiological deterioration may therefore represent a clinically actionable signal for closer surveillance, treatment adaptation, and supportive care optimization.

Third, our findings support a broader paradigm of precision supportive care aimed at preserving physiological resilience during systemic therapy. Potential strategies include individualized nutritional support, exercise-based rehabilitation, and interventions targeting metabolic and inflammatory dysregulation. Preserving physical function and treatment tolerance may represent an important complementary objective alongside tumor control.

Finally, the mediation analyses suggest that early physiological deterioration may represent a potential pathway linking treatment intensification and subsequent mortality risk. Although these findings should not be interpreted as definitive evidence of causality, they raise the possibility that modifying adverse physiological trajectories could improve treatment tolerability and potentially influence long-term outcomes. Future prospective studies are warranted to determine whether dynamic physiological monitoring and targeted supportive interventions can translate into meaningful clinical benefits for patients with advanced NSCLC.

### 4.4 Limitations

Several limitations should be considered when interpreting the present findings.

First, this was a retrospective observational study, and residual confounding cannot be completely excluded despite the use of IPTW, doubly robust estimation, landmark analyses, and extensive sensitivity analyses. Unmeasured factors, including frailty, socioeconomic status, treatment adherence, and clinician-driven treatment selection, may have influenced both physiological trajectories and survival outcomes. Therefore, the observed associations should not be interpreted as definitive evidence of causality.

Second, although the 12-week landmark design standardized the assessment of early physiological changes and reduced reverse causation, it excluded patients who died, progressed rapidly, or became unevaluable before the landmark time point. This approach may have introduced survivor and selection biases and may limit the generalizability of the findings to the most vulnerable patients with advanced NSCLC.

Third, physiological assessment relied primarily on single-slice CT measurements at the L3 vertebral level and conventional functional parameters. Although widely validated in oncology, these measures may not fully capture muscle quality, regional muscle heterogeneity, or other dimensions of physiological reserve. In addition, physiological trajectories were assessed over a relatively short interval, and measurement error cannot be entirely excluded. Furthermore, the optimal definition of low muscle mass in oncology remains uncertain. We adopted the AWGS 2019 criteria because they are widely used and appropriate for Asian populations; however, accumulating evidence suggests that disease-specific and treatment-specific skeletal muscle thresholds may provide superior prognostic discrimination in patients with cancer. For example, Gumustepe et al. recently demonstrated that prognostically relevant low muscle mass cut-offs in stage III NSCLC treated with definitive chemoradiotherapy differed from conventional consensus definitions (39). Therefore, the applicability of universal sarcopenia thresholds to patients with advanced NSCLC receiving immunotherapy-based treatment remains uncertain and warrants further investigation.

Fourth, advanced NSCLC is characterized by substantial biological heterogeneity. Comprehensive molecular information beyond PD-L1 expression, including co-occurring genomic alterations, tumor mutational burden, and other biomarkers associated with immunotherapy response, was not uniformly available. Consequently, the identified physiological phenotypes may partially reflect underlying biological heterogeneity that could not be fully accounted for in the present analyses.

Fifth, the proposed physiological phenotypes were derived and internally validated within a single-center cohort and have not undergone external validation in independent populations or alternative therapeutic settings. Their reproducibility and generalizability therefore remain uncertain. Moreover, because these phenotypes were generated using an unsupervised data-driven approach, they should be regarded as exploratory classifications rather than formal diagnostic entities analogous to EWGSOP2 or AWGS 2019. No standardized diagnostic criteria or predefined cut-off values currently exist for phenotype assignment.

Sixth, early physiological deterioration in advanced NSCLC is likely multifactorial. Functional decline may result from treatment-related toxicity but may also reflect disease progression, tumor burden, systemic inflammation, cachexia, intercurrent illness, medication exposure (including corticosteroid use), and other patient-specific factors. Although we adjusted for baseline disease burden, inflammatory status, and performance status and implemented a 12-week landmark framework, the retrospective design precludes definitive separation of treatment-associated physiological decline from deterioration related to the underlying disease process. Therefore, the identified physiological phenotypes should be interpreted as representing the combined influences of treatment exposure, disease progression, tumor biology, and host vulnerability rather than purely treatment-induced processes.

Finally, the counterfactual mediation analyses rely on assumptions that cannot be fully verified in observational studies, particularly the assumptions of no unmeasured exposure–mediator and mediator–outcome confounding and correct model specification. Therefore, the estimated indirect associations should be interpreted as potential explanatory pathways rather than precise estimates of causal mediation.

Despite these limitations, the present study provides a comprehensive longitudinal characterization of treatment-related physiological heterogeneity in advanced NSCLC and generates testable hypotheses regarding the potential associations between early physiological deterioration and long-term outcomes. Future prospective multicenter studies with external validation and richer molecular profiling are warranted to determine whether dynamic physiological phenotyping can improve risk stratification and guide individualized supportive interventions in patients receiving systemic therapy.

## 5 Conclusions

In conclusion, early treatment-related physiological deterioration in advanced NSCLC is highly heterogeneous and can be summarized into reproducible data-driven physiological phenotypes with distinct prognostic implications. The functional collapse phenotype identifies a particularly vulnerable subgroup at increased risk of mortality and provides prognostic information beyond conventional radiographic response assessments.

Our findings further suggest that early physiological attrition may represent an important potential explanatory pathway associated with subsequent survival outcomes following treatment intensification. These results highlight the value of longitudinal physiological phenotyping for improving risk stratification and understanding treatment heterogeneity in advanced NSCLC.

The integration of dynamic physiological monitoring into routine care may facilitate the early identification of patients at risk of treatment-related decompensation and inform individualized supportive interventions aimed at preserving physiological resilience. Future prospective multicenter studies are warranted to externally validate these findings and determine whether modifying adverse physiological trajectories can improve long-term outcomes.

Preserving physiological resilience may therefore represent an important complementary therapeutic objective alongside tumor control in the management of advanced NSCLC.

## Data Availability

The raw data supporting the conclusions of this article will be made available by the authors, without undue reservation.
