# Supplementary material for: Early physiological phenotypes and survival outcomes in advanced non-small cell lung cancer: a longitudinal study of dynamic muscle loss and functional decline
Source: Front Oncol. 2026 Aug 14;16:1882281. doi: 10.3389/fonc.2026.1882281 (PMC13522817; doi:10.3389/fonc.2026.1882281)
Supplement: Supplementary file 1 [file DataSheet1.docx]

**Early Physiological Phenotypes and Survival Outcomes in Advanced Non-Small Cell Lung Cancer: A Longitudinal Study of Dynamic Muscle Loss and Functional Decline**

**Supplementary Material**

**Figure S1.** Propensity score weighting diagnostics and sensitivity analyses for the IPTW cohort.

**(A)** Love plot showing the absolute SMDs of all baseline covariates included in the propensity score model before (blue circles) and after (orange circles) PTW. The dashed vertical line indicates the prespecified threshold of |SMD| = 0.10 for adequate covariate balance.

**(B)** Propensity score overlap displaying the kernel density distributions of propensity scores in the Monotherapy and Combination groups before weighting, demonstrating sufficient overlap and supporting the positivity assumption.

**(C)** Distribution of stabilized IPTW weights showing the frequency distribution of individual weights and assessing the presence of extreme weights.

**(D)** ESScomparing the original sample sizes and the corresponding ESS after weighting for both treatment groups.

**(E)** Weight trimming sensitivity analyses evaluating the robustness of the estimated treatment effect after applying alternative weight truncation strategies (untrimmed, 1st–99th percentile trimming, and 5th–95th percentile trimming).

**(F)** Alternative weighting analyses presenting HRs and 95% confidence intervals derived from multiple analytical approaches, including multivariable Cox regression, doubly robust estimation, overlap weighting, and standard IPTW.

Baseline covariates included in the propensity score model were age, sex, body mass index, smoking history, histological subtype, PD-L1 tumor proportion score, ECOG performance status, number of metastatic organs, liver metastasis, bone metastasis, brain metastasis, adrenal metastasis, albumin, C-reactive protein, neutrophil-to-lymphocyte ratio, skeletal muscle index, muscle attenuation, baseline sarcopenia, and baseline corticosteroid exposure.

**Abbreviations:** **CI**, confidence interval; **ESS**, effective sample size; **HR**, hazard ratio; **IPTW**, inverse probability of treatment weighting; **PD-L1**, programmed death-ligand 1; **SMD**, standardized mean difference

**Figure S2.** Unsupervised consensus clustering diagnostics for optimal cluster-number selection.

**(A)** Average silhouette width analysis for candidate cluster numbers (K = 2 – 6). The highest average silhouette width was observed at K = 3 (silhouette width = 0.58), indicating the best balance between within-cluster cohesion and between-cluster separation.

**(B)** CDF curves across different numbers of clusters (K = 2 – 6). The CDF distribution demonstrated increased stability and reduced incremental gain with increasing K, with K = 3 showing a stable consensus structure.

**(C)** Relative change in the area under the CDF curve (ΔCDF). The relative increase in CDF area decreased substantially after K = 3, suggesting limited additional structural information gained from further partitioning. Collectively, these analyses supported K = 3 as the optimal and most parsimonious clustering solution.

**Table S1**. Operational definitions and clinical interpretation of the three data-driven early physiological phenotypes identified by unsupervised hierarchical clustering

| Characteristic | P1 (Stable) | P2 (Wasting) | P3 (Collapse) |
| --- | --- | --- | --- |
| ΔSMI | Minimal decline | Moderate-to-severe decline | Moderate decline |
| ΔSMD (HU) | Preserved | Mild-to-moderate reduction | Marked reduction |
| ΔHandgrip strength | Preserved | Moderate reduction | Marked reduction |
| ΔGait speed | Preserved | Relatively preserved | Marked reduction |
| ΔECOG PS | Stable | Mild deterioration | Pronounced deterioration |
| Dominant physiological pattern | Global physiological stability | Predominant skeletal muscle wasting with relatively preserved physical function | Concurrent functional deterioration and performance status collapse |
| Clinical interpretation | Physiological resilience | Muscle wasting phenotype | Physiological collapse phenotype |

**Note:** The three phenotypes were derived using unsupervised hierarchical clustering based on standardized 12-week changes in ΔSMI, ΔSMD, handgrip strength, gait speed, and ΔECOG PS. Because the clusters were generated using a data-driven approach, no predefined diagnostic thresholds or cut-off values were imposed for phenotype assignment. These categories should be regarded as exploratory, hypothesis-generating classifications intended to characterize heterogeneous physiological responses during treatment and require external validation before standardized diagnostic criteria can be established.

**Abbreviations:** SMI, skeletal muscle index; SMD, skeletal muscle density; ECOG PS, Eastern Cooperative Oncology Group Performance Status.

**Table S2.** Clustering diagnostics and stability metrics for cluster-number selection (K = 2 to 6‬).

| Number of clusters (K) | Average silhouette width | Mean Jaccard similarity coefficient | Clinical interpretability | Final decision |
| --- | --- | --- | --- | --- |
| 2 | 0.49 | 0.87 | Moderate | No |
| 3 (Selected) | 0.58 | 0.84 | Excellent | Yes |
| 4 | 0.52 | 0.71 | Poor | No |
| 5 | 0.46 | 0.66 | Poor | No |
| 6 | 0.39 | 0.6 | Poor | No |

**Note:** The mean Jaccard similarity coefficient was calculated using non-parametric bootstrap resampling with 1,000 iterations. A Jaccard coefficient ‭> 0.75‬ was predefined as the threshold for robust cluster reproducibilit**y.**

**Table S3.** Interaction Between Treatment Regimen and Early Physiological Phenotypes on Treatment Tolerability and Short-Term Antitumor Activity in the IPTW-Weighted Cohort

| Outcomes | Monotherapy | | Combination Therapy | | *P*_interaction_ |
| --- | --- | --- | --- | --- | --- |
|  | P1 (Stable) | P2/P3 (Vulnerable) | P1 (Stable) | P2/P3 (Vulnerable) |  |
| Treatment tolerability |  |  |  |  |  |
| Overall Grade ≥3 adverse events, n (%) | 12.40% | 24.50% | 18.20% | 45.90% | 0.002 |
| Hematologic toxicity, n (%) | 2.10% | 4.90% | 15.40% | 38.20% | <0.001 |
| Immune-related adverse events, n (%) | 8.50% | 18.20% | 6.20% | 12.10% | 0.142 |
| Permanent treatment discontinuation, n (%) | 5.20% | 15.10% | 10.40% | 28.50% | 0.015 |
| Short-term antitumor activity |  |  |  |  |  |
| Objective response rate (ORR), % | 18.30% | 12.80% | 36.80% | 20.20% | 0.011 |
| Disease control rate (DCR), % | 62.10% | 48.90% | 79.00% | 60.30% | 0.018 |

**Notes:** Values are presented as weighted percentages derived from the IPTW pseudo-population. Physiological phenotypes were classified according to the 12-week data-driven clustering results. Because of the limited sample size and event numbers within individual vulnerable phenotypes, Phenotype 2 (Wasting) and Phenotype 3 (Collapse) were combined for interaction analyses. Interaction P values were obtained from weighted logistic regression models including treatment-by-phenotype interaction terms.

**Abbreviations: DCR**, disease control rate; **irAEs**, immune-related adverse events; **IPTW**, inverse probability of treatment weighting; **ORR**, objective response rate; **P1**, Stable phenotype; **P2**, Wasting phenotype; **P3**, Collapse phenotype.

**Table S4.** Associations of Early Physiological Phenotypes With Subsequent Clinical Consequences Across RECIST Response Categories in the IPTW-Weighted Cohort

| Outcome | CR/PR | SD | PD | *P*_interaction_ |
| --- | --- | --- | --- | --- |
| Persistent muscle atrophy  (SMI decline ≥10%) |  |  |  |  |
| P2 vs. P1, OR (95% CI) | 1.84 (1.05–3.22) | 1.91 (1.18–3.10) | 1.76 (0.98–3.15) | 0.82 |
| P3 vs. P1, OR (95% CI) | 2.45 (1.34–4.49) | 2.71 (1.58–4.65) | 2.29 (1.24–4.21) | 0.76 |
| Incident cachexia |  |  |  |  |
| P2 vs. P1, OR (95% CI) | 1.68 (0.97–2.91) | 1.82 (1.10–3.00) | 1.59 (0.89–2.84) | 0.85 |
| P3 vs. P1, OR (95% CI) | 2.38 (1.31–4.32) | 2.64 (1.52–4.58) | 2.19 (1.17–4.08) | 0.73 |
| Grade ≥3 adverse events |  |  |  |  |
| P2 vs. P1, OR (95% CI) | 1.79 (1.05–3.05) | 1.86 (1.16–2.98) | 1.71 (0.96–3.05) | 0.88 |
| P3 vs. P1, OR (95% CI) | 2.65 (1.49–4.73) | 2.84 (1.65–4.90) | 2.51 (1.35–4.66) | 0.79 |
| Permanent treatment discontinuation |  |  |  |  |
| P2 vs. P1, OR (95% CI) | 1.72 (0.92–3.22) | 1.88 (1.04–3.39) | 1.66 (0.84–3.28) | 0.84 |
| P3 vs. P1, OR (95% CI) | 2.44 (1.27–4.70) | 2.69 (1.48–4.91) | 2.33 (1.17–4.64) | 0.77 |

**Notes:** Data are presented as ORs with corresponding 95% CIs, using Phenotype 1 (P1, Stable) as the reference category. RECIST response categories were defined according to the best overall response during treatment and classified as CR/PR, SD, and PD. Analyses were performed within the IPTW-weighted pseudo-population.

Interaction P values were derived from weighted logistic regression models incorporating phenotype-by-RECIST interaction terms. The absence of statistically significant interactions indicates that the associations between early physiological phenotypes and subsequent adverse clinical outcomes were generally consistent across different tumor response categories.

Persistent muscle atrophy was defined as a sustained decline in SMI of ≥10% from baseline. Cachexia was defined according to international consensus criteria.

**Abbreviations: CI**, confidence interval; **CR**, complete response; **OR**, odds ratio; **PD**, progressive disease; **P1**, Phenotype 1 (Stable); **P2**, Phenotype 2 (Wasting); **P3**, Phenotype 3 (Collapse); **PR**, partial response; **RECIST**, Response Evaluation Criteria in Solid Tumors; **SD**, stable disease; **SMI**, skeletal muscle index.

**Table S5.** Sensitivity Analyses Evaluating the Independent Associations of Early Physiological Phenotypes After Additional Adjustment for Tumor Response and Clinical Confounders

| Outcome | Model 1  OR (95% CI) | *P_value_* | Model 2  OR (95% CI) | *P_value_* |
| --- | --- | --- | --- | --- |
| Persistent muscle atrophy (SMI decline ≥10%) |  |  |  |  |
| P2 vs. P1 | 1.86 (1.23–2.82) | 0.003 | 1.73 (1.12–2.67) | 0.014 |
| P3 vs. P1 | 2.64 (1.74–4.00) | <0.001 | 2.41 (1.56–3.72) | <0.001 |
| Incident cachexia |  |  |  |  |
| P2 vs. P1 | 1.79 (1.17–2.75) | 0.008 | 1.65 (1.06–2.57) | 0.027 |
| P3 vs. P1 | 2.51 (1.64–3.86) | <0.001 | 2.28 (1.46–3.56) | <0.001 |
| Grade ≥3 adverse events |  |  |  |  |
| P2 vs. P1 | 1.82 (1.21–2.74) | 0.004 | 1.69 (1.11–2.58) | 0.016 |
| P3 vs. P1 | 2.72 (1.80–4.11) | <0.001 | 2.46 (1.60–3.79) | <0.001 |
| Permanent treatment discontinuation |  |  |  |  |
| P2 vs. P1 | 1.71 (1.05–2.79) | 0.031 | 1.58 (0.95–2.62) | 0.078 |
| P3 vs. P1 | 2.48 (1.52–4.04) | <0.001 | 2.21 (1.33–3.67) | 0.002 |

**Notes:** Data are presented as ORs with corresponding 95% CIs, using Phenotype 1 (P1, Stable) as the reference category. All analyses were conducted within the IPTW-weighted pseudo-population.

Model 1 was adjusted for age, sex, body mass index, ECOG performance status, histological subtype, number of metastatic organs, liver metastasis, PD-L1 TPS, baseline albumin concentration, baseline NLR, and treatment regimen.

Model 2 was additionally adjusted for best RECIST response category (CR/PR, SD, or PD), documented infection during the 12-week landmark period, systemic corticosteroid use, and baseline cachexia status.

The persistence of significant associations after sequential adjustment indicates that the identified physiological phenotypes capture clinically meaningful vulnerability beyond tumor response alone and are unlikely to merely represent surrogate markers of disease progression. These findings support the independent clinical relevance of the proposed early physiological phenotyping framework.

**Abbreviations:** **CI**, confidence interval; **CR**, complete response; **ECOG PS**, Eastern Cooperative Oncology Group performance status; **NLR**, neutrophil-to-lymphocyte ratio; **OR**, odds ratio; **P1**, Phenotype 1 (Stable); **P2**, Phenotype 2 (Wasting); **P3**, Phenotype 3 (Collapse); **PD**, progressive disease; **PD-L1 TPS**, programmed death-ligand 1 tumor proportion score; **PR**, partial response; **RECIST**, Response Evaluation Criteria in Solid Tumors; **SD**, stable disease.

**Table S6.** Incremental Performance of Sequential Landmark Cox Models for Subsequent Overall Survival

| Metric | Model 1 | Model 2 | Model 3 |
| --- | --- | --- | --- |
| Harrell’s C-index | 0.654 | 0.782 | 0.804 |
| ΔC-index | Reference | 0.128 | 0.022 |
| Likelihood ratio test | — | <0.001 vs. Model 1 | 0.004 vs. Model 2 |
| AIC | 1268.4 | 1214.8 | 1207.2 |

**Notes:** Three sequential landmark Cox proportional hazards models were constructed to evaluate the incremental prognostic value of early physiological phenotypes identified at the 12-week assessment.

Model 1 included baseline demographic and clinical characteristics only, including age, sex, BMI, baseline ECOG performance status, treatment regimen, and PD-L1 TPS.

Model 2 additionally incorporated the early physiological phenotypes (P2 [Wasting] and P3 [Collapse]) derived from the unsupervised clustering analyses.

Model 3 further included the interaction term between treatment regimen and Phenotype 3 (Collapse) to assess whether the prognostic implications of physiological collapse differed according to treatment strategy.

All survival analyses were initiated from the prespecified 12-week landmark to minimize immortal time bias, and only patients who remained alive and evaluable at the 12-week assessment were included in the analyses.

Model discrimination was evaluated using Harrell’s C-index. Incremental improvements in model performance were assessed using LRTs comparing nested models and the AIC, with lower AIC values indicating better overall model fit.

Because these analyses were exploratory and intended to assess the incremental prognostic utility of the proposed physiological phenotyping framework, the findings should be considered hypothesis-generating and warrant confirmation in independent external cohorts.

**Abbreviations:** AIC, Akaike information criterion; **BMI,** body mass index; **C-index**, concordance index; **ECOG PS**, Eastern Cooperative Oncology Group performance status; **LRT**, likelihood ratio test; **P2**, Wasting phenotype; **P3**, Collapse phenotype; **PD-L1 TPS**, programmed death-ligand 1 tumor proportion score.

**Table S7.** Counterfactual Mediation Analysis Evaluating the Potential Mediating Role of Physiological Phenotypes in the Association Between Treatment Regimen and Overall Survival

| Mediation Analysis Components | Potential Muscle-related Pathway (P2) | ***P _value_*** | Potential Functional Decline Pathway (P3) | ***P _value_*** |
| --- | --- | --- | --- | --- |
| Step 1. Path Coefficients (Log-transformed) |  |  |  |  |
| Path a: Treatment regimen → Phenotype | 0.795 (0.501–1.089) | <0.001 | 0.932 (0.642–1.222) | <0.001 |
| Path b: Phenotype → Grade ≥3 toxicity | 1.048 (0.670–1.426) | <0.001 | 1.265 (0.881–1.649) | <0.001 |
| Path c: Grade ≥3 toxicity → Overall survival (HR) | 1.954 (1.421–2.687) | <0.001 | 2.312 (1.684–3.174) | <0.001 |
| Step 2. Effect Decomposition (Bootstrapped) |  |  |  |  |
| TE | 0.485 (0.124–0.846) | 0.012 | 0.485 (0.124–0.846) | 0.012 |
| ADE | 0.321 (0.015–0.627) | 0.045 | 0.321 (0.015–0.627) | 0.045 |
| TIE | 0.164 (0.052–0.276) | 0.002 | 0.232 (0.094–0.370) | <0.001 |
| Step 3. Clinical Interpretation |  |  |  |  |
| Estimated Mediation Proportion (%)* | 33.81 | — | 47.84 | — |
| Potential Biological Interpretation | Treatment-related metabolic deterioration and skeletal muscle depletion | | Acute functional decline and treatment modification (RDI <80%) | |
| Step 4. Sensitivity Analysis |  |  |  |  |
| E-value for Indirect Association (Point Estimate) | 2.12 |  | 2.53 |  |
| E-value for 95% CI (Lower Limit) | 1.45 |  | 1.82 |  |

**Notes:**

(A) Mediation model specification: The exposure variable (X) was treatment regimen (combination therapy versus monotherapy); the mediator variable (M) was the longitudinal physiological phenotype (P2 or P3); and the outcome variable (Y) was OS.

(B) Mediation effects were estimated within a counterfactual mediation framework using the IPTW-weighted cohort. ADE, TIE, and TE were estimated using non-parametric bootstrap resampling (2,000 iterations).

(C) Mediation proportions represent the proportion of the observed association between treatment regimen and overall survival that may be statistically explained by the physiological phenotypes under the assumptions of the mediation model.

(D) E-values were calculated to evaluate the robustness of the estimated indirect associations to potential unmeasured confounding.

(E) The identified pathways should be interpreted as potential mediation pathways rather than definitive causal mechanisms because residual confounding cannot be completely excluded.

(*) Estimated mediation proportions were calculated separately for each pathway and should not be interpreted as additive components of the total effect because the indirect associations through different physiological phenotypes may partially overlap.

**Abbreviations: ADE**, Average Direct Effect; **CI**, Confidence Interval; **G3**, Grade 3; **HR**, Hazard Ratio; **IPTW**, Inverse Probability of Treatment Weighting; OS, Overall Survival; **RDI**, Relative Dose Intensity; **TE**, Total Effect; **TIE**, Total Indirect Effect.

**Table S8.** Counterfactual Decomposition of the Association Between Treatment Regimen and Overall Survival Through Potential Physiological Mediation Pathways

| Effect Type | Potential Pathway | Estimate (Log-HR) | 95% CI | *P _value_* |
| --- | --- | --- | --- | --- |
| TE | Combined association | 0.485 | 0.124 – 0.846 | 0.012 |
| ADE | Direct association | 0.321 | 0.015 – 0.627 | 0.045 |
| TIE | Via Phenotype P2 | 0.164 | 0.052 – 0.276 | 0.002 |
| TIE | Via Phenotype P3 | 0.232 | 0.094 – 0.370 | <0.001 |
| Combined Indirect Association† | P2 + P3 | 0.396 | 0.176 – 0.616 | <0.001 |

**Notes：**

(A) Mediation model specification: Treatment regimen (combination therapy versus monotherapy) was specified as the exposure (X), the 12-week physiological phenotypes (P2 and P3) as the potential mediators (M), and OS as the outcome (Y).

(B) All estimates were derived from inverse probability of treatment weighting (IPTW)-weighted counterfactual mediation models to minimize confounding arising from baseline imbalances between treatment groups.

(C) Effect estimates, 95% confidence intervals, and P values were obtained using non-parametric bootstrap resampling with 2,000 iterations.

(D) The indirect associations through P2 and P3 were estimated separately and may partially overlap. Therefore, the estimated mediation effects should not be interpreted as additive components of the total effect.

(E) The combined indirect association (†) represents the aggregate contribution of the identified physiological phenotypes to the observed association between treatment regimen and overall survival and should be interpreted as a potential explanatory pathway rather than definitive evidence of causality.

(F) A positive Log-HR indicates an increased mortality risk associated with the corresponding pathway.

**Abbreviations: ADE**, Average Direct Effect; **CI**, Confidence Interval; **IPTW**, Inverse Probability of Treatment Weighting; **Log-HR**, Log-transformed Hazard Ratio; **OS**, Overall Survival; **TE**, Total Effect; **TIE**, Total Indirect Effect.
